# Curating a longitudinal research resource using linked primary care EHR data—a UK Biobank case study

**DOI:** 10.1093/jamia/ocab260

**Published:** 2021-12-13

**Authors:** Philip Darke, Sophie Cassidy, Michael Catt, Roy Taylor, Paolo Missier, Jaume Bacardit

**Affiliations:** School of Computing, Newcastle University, Newcastle upon Tyne, UK; Central Clinical School, The University of Sydney, Sydney, Australia; Population Health Sciences Institute, Newcastle University, Newcastle upon Tyne, UK; Translational and Clinical Research Institute, Newcastle University, Newcastle upon Tyne, UK; School of Computing, Newcastle University, Newcastle upon Tyne, UK; School of Computing, Newcastle University, Newcastle upon Tyne, UK

**Keywords:** electronic health records, medical record linkage, longitudinal studies, phenotype, diabetes mellitus

## Abstract

Primary care EHR data are often of clinical importance to cohort studies however they require careful handling. Challenges include determining the periods during which EHR data were collected. Participants are typically censored when they deregister from a medical practice, however, cohort studies wish to follow participants longitudinally including those that change practice. Using UK Biobank as an exemplar, we developed methodology to infer continuous periods of data collection and maximize follow-up in longitudinal studies. This resulted in longer follow-up for around 40% of participants with multiple registration records (mean increase of 3.8 years from the first study visit). The approach did not sacrifice phenotyping accuracy when comparing agreement between self-reported and EHR data. A diabetes mellitus case study illustrates how the algorithm supports longitudinal study design and provides further validation. We use UK Biobank data, however, the tools provided can be used for other conditions and studies with minimal alteration.

## INTRODUCTION

Access to nonemergency healthcare in the UK is overseen by General Practitioners (GPs). Electronic Health Records (EHRs) maintained by GPs have been used in observational research for over 30 years^1^ and are often used in longitudinal cohort studies, for example, to follow participants after the end of data collection.^2^^,^^3^ However, in contrast with the highly curated data collected under a study protocol, EHR data collection is unstandardized, driven by patient need, and subject to a range of biases.^4^ The successful integration of linked EHR data in cohort studies is therefore challenging but increasingly important in a wide variety of research fields.

Using UK Biobank^5^ as an exemplar, we developed methodology to incorporate linked primary care EHR data from multiple data providers within a cohort study. We infer periods of data collection in contrast to typical approaches that censor participants when they deregister from a medical practice. Around 40% of participants with multiple registration periods have longer follow-up under this approach without sacrificing phenotyping accuracy when comparing agreement between self-reported data for a range of conditions and medications.

A diabetes mellitus case study was used to demonstrate how the algorithm supports longitudinal study design. Two NHS-approved diabetes prediction tools performed in line with previous validation studies, further validating the approach. We also contribute extensive supplementary material examining the quality of linked EHR data in UK Biobank. R code is provided to enable researchers to apply the approach to UK Biobank and other cohort studies with linked EHR data.

## STUDY DATA

UK Biobank is a large prospective study of serious illness in middle and old age with longitudinal follow-up achieved primarily through linkage to national data sets.^6^ Interim primary care EHR data were released in September 2019 covering around 230 000 participants with subsequent updates available for COVID-19-related research. These were obtained from intermediaries including the suppliers of GP practice management systems and were linked and de-identified by UK Biobank.^7^ Participants provided written consent.

Data were recorded by healthcare professionals working at GP practices in England, Scotland, and Wales as part of routine patient care. The interim release included GP practice registration periods, coded diagnoses, test results, drug prescriptions, and administrative data recorded prior to 2016/17. UK Biobank purposefully carried out minimal data cleaning prior to release^7^ and the volume of data available varied considerably across individuals.

## CURATING A LONGITUDINAL RESEARCH RESOURCE

The successful integration of linked EHR data required: 1) cleaning and validating the raw data, 2) identifying periods of data collection relative to study visits, and 3) extracting clinically relevant diagnoses, observations, and test results.

### Initial data cleaning

Data were provided from the TPP SystmOne, EMIS Web, and Vision practice management systems, in contrast with large UK EHR repositories which typically use data from a single system. Registration period, clinical event, and prescription record quality were assessed against standards developed from the Clinical Practice Research Datalink “acceptable patient flag”^8^ (Supplementary Table S1). Data quality varied by provider (Supplementary Tables S2–S5). Records with missing dates or codes were excluded.

### Identifying periods of EHR data collection

**Figure 1.**
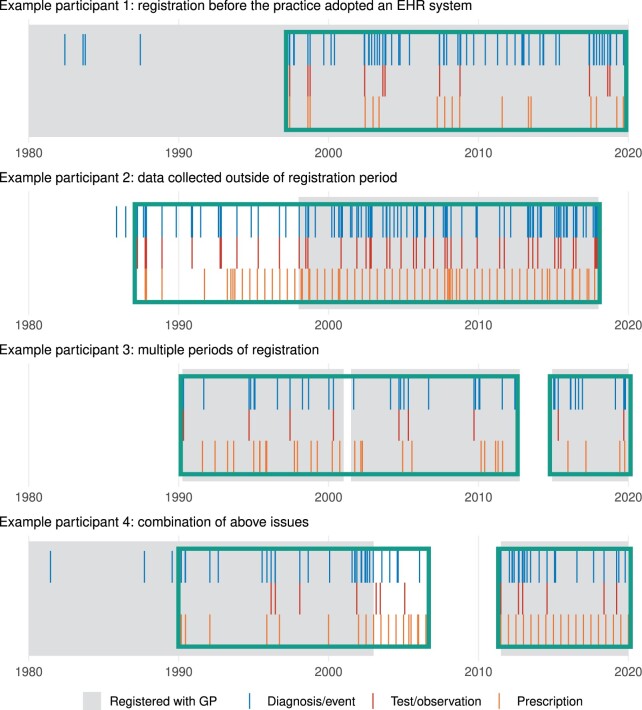
Common issues in EHR data collection illustrated with synthetic participant data. These resemble realistic participant types, for example, around 70% of UK Biobank participants have data outside of periods of practice registration. Example 1—individual registered with a practice at birth that subsequently adopted an EHR system in the 1990s (prior records are paper-based). Example 2—individual registered with a practice in 1999 but records are also held from a previous period of registration with another practice. Example 3—multiple periods of registration are available from different practices and/or data providers. Example 4—a combination of the above issues. The boxed areas illustrate the inferred periods of data collection using our algorithm.

Understanding when data have been collected is essential for longitudinal studies. Existing primary care EHR research often focusses on data recorded after the introduction of the National Health Service (NHS) Quality and Outcomes Framework (QOF) in 2004 which encouraged consistent recording practices across a range of conditions. Practice registration records are typically used determine periods of data collection, for example, by selecting from participants registered with a GP at study start and censoring at practice deregistration. There are limitations when applying this approach to linked EHR data. GP practices began to adopt EHR systems in the 1980s and data recorded prior to the QOF may have clinical importance; while participants may only register with a single NHS practice at a time, records may follow individuals that transfer between practices resulting in the presence of data outside of registration periods; and censoring participants at the first practice deregistration may curtail follow-up (Figure  1). The latter is of particular concern for cohort studies, where a natural objective is to leverage EHR data to follow participants over time.

**Figure 2.**
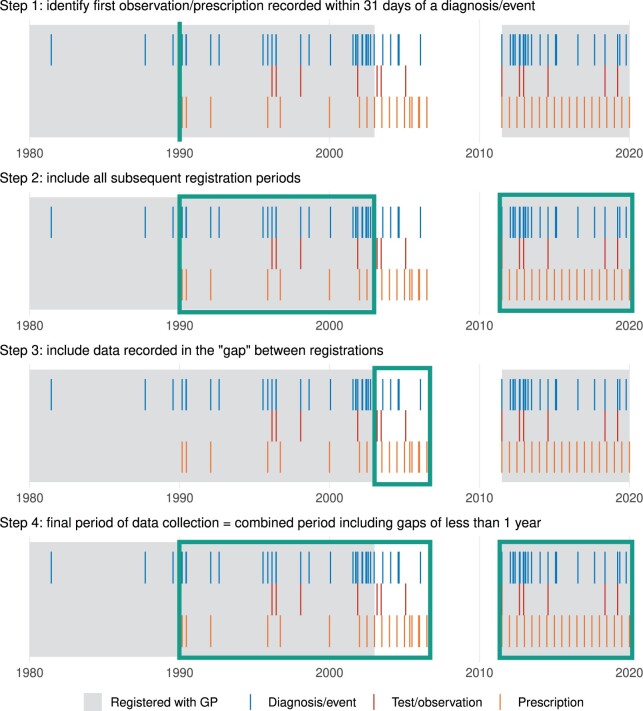
Application of our algorithm to determine periods of complete EHR data collection. The example participant has multiple periods of registration and data outside of registration periods. The boxed areas are the inferred periods of data collection. Further details are included in the Supplementary Materials (Algorithm A1 and Supplementary Figure S1).

To address these limitations, an algorithm was developed to identify periods of EHR data collection across practice registration periods (exemplified in Figure  2). Full details are included in Section 2 in the Supplementary Material. A high-level description of the algorithm follows:

1. The period of data collection starts at the first observation/prescription record accompanied by a diagnosis/clinical event record, for example, to capture the recording of a BMI when joining a medical practice.
2. Collection was assumed to be complete until practice deregistration and during any subsequent registration periods.
3. Periods of record collection outside of registration periods were included if they contained at least one nonprescription record. Collection was assumed to have taken place during unregistered periods shorter than 1 year.
4. Participants were censored at the earlier of the inferred end of data collection, the data extract date, or the date of death in linked death registry data.

The algorithm is applied separately for each data provider and the resultant periods combined.

### Extracting clinically relevant data from linked EHRs

UK Biobank data feature a range of coding classifications and multiple data fields for observations and biomarkers^7^^,^^9^ that must be handled. Existing rule-based phenotyping algorithms aim to replicate diagnostic criteria^10^ using clinical code sets to identify relevant exposures and outcomes,^11^ however, code set repositories for UK EHR research^2^^,^^12^^,^^13^ typically only cover Read v2 diagnostic codes and limited prescription coding. Comprehensive Read v2, Clinical Terms Version 3 (CTV3), and British National Formulary code sets covering a range of conditions, observations, biomarkers, and drugs were developed (Section 7 in Supplementary Material). Units of measurement were rarely provided in the data, and code descriptions and data dictionaries^14^ were often unreliable. An approach was therefore developed to harmonize units (Section 3.2 in Supplementary Material).

## VALIDATING THE PROCESSED EHR DATA

### Assessing the algorithm against GP registration records

A typical approach is to assume full data collection during periods of GP registration. For example, 191 878 participants (83.4% of participants with clinical event data) were registered with a GP at the first UK Biobank study visit with a mean period of 6.9 years to practice deregistration. In contrast, our algorithm maximized study population and follow-up, identifying 196 901 (85.6%) participants with active data collection at the first visit and a mean follow-up of 7.4 years. By design, no participant had a shorter follow-up period under the algorithm. The impact varied by participant, however, the synthetic examples in Figure  1 represent common scenarios:

#### Participant 1

GP registration occurs before the inferred start of data collection for 67% of participants. The mean period of registration before data collection starts is 11.2 years (median 5.8 years). The date of GP registration is often a poor indicator of the start of data collection.

#### Participant 2

Conversely, 24% of participants show evidence of data collection before the first GP registration (mean 5.8 years of additional data). This may be the result of data transfer when participants move between GP practices. Studies that identify this additional data may be able to use earlier study start dates for example.

#### Participant 3

About 31% of participants have multiple registration periods (Supplementary Table S5). Around 40% of these participants have a longer follow-up under our algorithm (mean 3.8 years). As additional linked EHR data are published, the number of participants with multiple periods of registration will increase and methods that follow participants across registrations will be required to maintain follow-up.

### Agreement with self-reported medical conditions and medication

While “ground truth” medical state is typically unavailable, results can be compared with self-reported health in UK Biobank. Participants were phenotyped for selected conditions using EHR data and the results compared with self-reported health at the first study visit (Table 1 and Section 4 in Supplementary Material). The comparison was made for participants with at least 1 year of continuous data collection determined using: 1) our algorithm and 2) assuming data collection only during periods of GP registration. The algorithm generally showed better sensitivity for conditions however the difference between approaches was small. The algorithm therefore maximized study population and follow-up without sacrificing phenotyping accuracy.

**Table 1.** Agreement between self-reported and EHR data at the first UK Biobank visit for the conditions and medications used in the QDiabetes-2018 model^15^

Active data collection at first UK Biobank visit determined using:	Our algorithm (Algorithm A1 in Supplementary Materials)			GP registration records		
	Sensitivity	Specificity	Precision	Sensitivity	Specificity	Precision
Presence of a previous diagnostic record						
Diabetes	**94.4**	99.8	95.8	94.3	99.8	**95.9**
Hypertension	**72.2**	98.1	93.2	72.1	98.1	**93.3**
MI/heart attack	70.6	99.9	94.7	**70.7**	99.9	**94.8**
Angina	59.8	99.4	76.4	**59.9**	99.4	**76.5**
Stroke	**55.8**	99.6	**65.2**	55.4	99.6	64.9
Transient ischemic attack	**56.0**	99.4	23.7	55.8	99.4	23.7
Bipolar disorder	**67.2**	99.7	41.3	67.1	**99.8**	**41.8**
Schizophrenia	**87.4**	99.8	28.4	87.0	99.8	**29.1**
Polycystic ovarian syndrome	**57.3**	99.8	22.4	57.0	99.8	**23.0**
Presence of a prescription record in previous 90 days						
Antihypertensives	86.0	98.2	93.6	**86.2**	98.2	**93.7**
Statins	88.1	97.9	89.0	**88.2**	97.9	89.0
Corticosteroids	49.6	99.3	45.1	**49.8**	99.3	**45.2**
Atypical antipsychotics	79.7	100.0	85.6	**80.4**	100.0	85.6

*Note:* Agreement was defined as the presence of a diagnostic record prior to the visit for medical conditions, or the presence of a prescription record in the 90 days prior to the visit for current medication. *Sensitivity* is the proportion of self-reporting participants that have a confirmatory EHR record. *Specificity* is the proportion of participants that do not self-report that also do not have an EHR record. *Precision* is the proportion of participants with an EHR record that also self-report. Overall agreement was similar under each approach, indicating that the algorithm did not sacrifice phenotyping accuracy. Bold indicates higher value.

The metrics in Table  1 are driven by provider 3 (England TPP) which supplied the majority of linked EHR data. Performance by data provider is provided in Supplementary Tables S8–S11. The algorithm outperformed the use of registration records for provider 1 (England Vision) where the primary issue is identifying the start of data collection. Performance was similar for the remaining providers which featured registration periods that conflict or have gaps suggesting that the algorithm handles these cases well.

Agreement between self-reported and EHR data varied by condition (Section 4 in Supplementary Material). Prescription data appeared to be of lower quality, with evidence of missing or truncated prescription histories beyond the “system-wide block of missing [provider 2 (Scotland) prescription] records prior to 2012”.^7^ UK Biobank prescription data also features nonstandard coding complicating its use (Section 3.3 in Supplementary Material). Accordingly, agreement with self-reported data was generally lower, however, this may also be the result of prescriptions made outside of primary care (eg, emergency corticosteroids) or inconsistent self-reporting.

## CASE STUDY: LONGITUDINAL DIABETES PHENOTYPING

**Figure 3.**
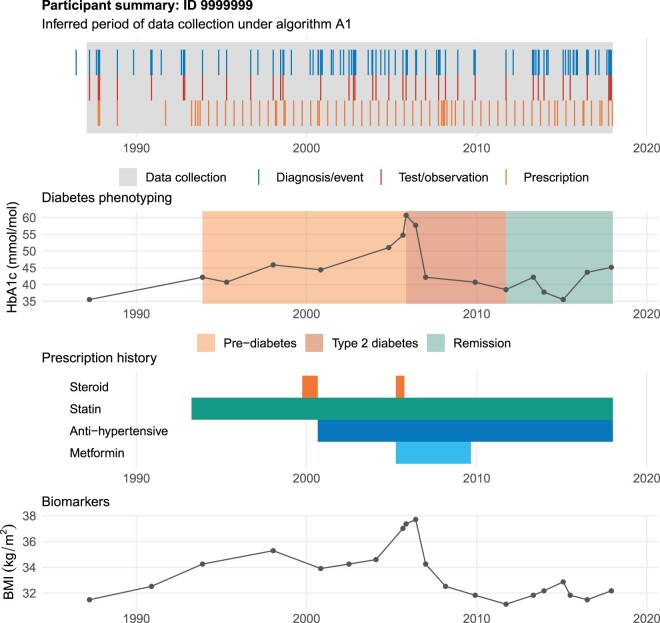
Example output from the longitudinal phenotyping tool for a synthetic participant. Our algorithm was used to identify periods of complete data collection (top panel). Periods of nondiabetic hyperglycemia (prediabetes), type 2 diabetes, and remission were identified. Periods of medication and biomarkers are also shown. We phenotyped periods of complete data collection to reduce the risk of inaccurately identifying the date of incidence of diabetes. Similar phenotyping approaches using linked EHR data can be used to enforce study criteria or identify more complex endpoints.

To demonstrate how our approach supports study design, participants were longitudinally phenotyped for health states associated with diabetes mellitus (diabetes). Diabetes was selected as its diagnosis and management typically takes place in a primary care setting. Diabetes subtypes differ markedly in clinical features but are not widely self-reported in UK Biobank. Previous approaches estimated subtype from self-reported data,^16^ however, the newly available linked EHR data potentially offer more objective supporting information. The phenotyping approach was developed with clinical experts (Section 5 in Supplementary Material) but can be readily adapted for other conditions. Figure  3 shows phenotyping tool output for a synthetic participant.

Longitudinal phenotyping is challenging for chronic conditions as multiple diagnosis codes are often recorded over time, for example, at annual care reviews. The first instance of a code may not correspond to the date of diagnosis if data collection is incomplete. We aimed to minimize the risk of misidentifying the date of incidence by restricting phenotyping to periods of complete data collection as inferred by our algorithm.

To further validate our algorithm, the performance of two NHS-approved diabetes prediction tools was evaluated on the processed data. QDiabetes-2018^15^ and the Leicester Risk Assessment score^17^ are used to triage individuals at risk of diabetes.^18^ QDiabetes was developed using primary care EHR data and results are shown in Table  2. Leicester score results are presented in Section 6.2 of the Supplementary Materials. Both scores performed broadly in line with previous validation studies, indicating that the data processed is suitable for use in longitudinal diabetes studies. This reinforces recent work suggesting that risk factor associations in UK Biobank are generalizable across a range of conditions^19^ despite the healthier, less-deprived, and less diverse ethnic make-up relative to the UK population.^20^

**Table 2.** QDiabetes-2018 model performance (concordance index) for the 10-year incidence of diabetes using UK Biobank EHR data

	Model A (demographic data, medical history, and BMI)	Model B (A plus current fasting plasma glucose result)	Model C (A plus current HbA1c result)
Male			
UK Biobank	0.781	0.831	0.882
QResearch^15^	0.814	0.866	0.855
Female			
UK Biobank	0.832	0.877	0.904
QResearch^15^	0.834	0.889	0.878

*Note:* Performance on the integrated linked EHR data is broadly in line with Hippisley-Cox et al.^15^ (shown as QResearch).

## CONCLUSION

Linked EHR data can be a valuable source of data to cohort studies. An approach was presented to integrate linked primary care EHR data within a cohort study. This maximizes study populations and follow-up using a rule-based approach to determine periods of EHR data collection for each participant. The processed UK Biobank EHR data showed good agreement with self-reported health status and NHS-approved diabetes prediction tools performed well in a longitudinal study, validating the approach and demonstrating how linked EHR data can be used in study design.

We provide extensive supplementary material examining the quality of linked EHR data in UK Biobank. Tools are also provided to implement our approach. These are designed to be general-purpose and support a range of study designs. The approach generalizes to other medical conditions and studies using linked EHR data.

## FUNDING

This work was supported by the Engineering and Physical Sciences Research Council, Centre for Doctoral Training in Cloud Computing for Big Data, Newcastle University (grant number EP/L015358/1).

## AUTHOR CONTRIBUTIONS

PD conceptualized the work, generated the code sets, processed the data, analyzed the results, drafted the manuscript, and is the guarantor. SC and RT provided diabetes-specific input and reviewed the clinical code sets. JB and PM supervised the research. All authors critically revised the manuscript and approved the final version.

## SUPPLEMENTARY MATERIAL

Supplementary material is available at *Journal of the American Medical Informatics Association* online. R code including participant plotting tools are available at https://github.com/philipdarke/ukbb-ehr-data.

## Supplementary Material

ocab260_Supplementary_Data
