# Supplementary material for: Curating a longitudinal research resource using linked primary care EHR data—a UK Biobank case study
Source: J Am Med Inform Assoc. 2021 Dec 13;29(3):546–52. doi: 10.1093/jamia/ocab260 (PMC8800530; doi:10.1093/jamia/ocab260)
Supplement: ocab260_Supplementary_Data [file ocab260_supplementary_data.pdf]

Curating a longitudinal research resource using linked primary care EHR data – a UK Biobank case study
Supplementary materials

`R` code including participant plotting tools are available at https://github.com/philipdarke/ukbb-ehr-data.

CONTENTS

1 Initial data cleaning **2**

2 Identifying periods of EHR data collection **6**

3 Extracting clinically relevant data from linked EHRs **12**

3.1 Summary of approach 12

3.2 Unit harmonisation 12

3.3 Prescription data 14

4 Validating the processed EHR data **17**

5 Longitudinal diabetes phenotyping **21**

5.1 Code set development 21

5.2 Diabetes incidence criteria 21

5.3 Diabetes remission criteria 21

5.4 Pre–diabetes criteria 22

6 Performance of NHS–approved diabetes risk prediction tools **22**

6.1 QDiabetes–2018 22

6.2 Leicester risk score 29

7 EHR code sets 32

7.1 Diabetes diagnosis and related coding . . . 32

7.2 Hypertension diagnosis . . . 33

7.3 Cardiovascular disease diagnosis . . . 33

7.4 Other conditions . . . 35

7.5 Anthropometric . . . 36

7.6 Smoking status . . . 37

7.7 Blood glucose . . . 39

7.8 Drug prescriptions . . . 40

8 UK Biobank coding 43

1. INITIAL DATA CLEANING

Table S1 summarises data standards developed from the Clinical Practice Research Datalink (CPRD) Aurum "acceptable patient flag"[1]. Data quality was assessed against these standards by data provider. Coding completeness was also assessed and results are summarised in tables S2 to S4.

Records with missing dates (as defined in table S1) or codes were excluded. Dates were estimated where records had been de–identified by UK Biobank[2]. Birth–dated records (dated 02/02/1902) were set to the estimated date of birth[1]. Records recorded during the birth year (dated 03/03/1903) were assumed to have been recorded at age six months.

Data providers 1 to 3 had large numbers of registration records with missing de–registration dates. These were assumed to be open periods of registration at the date of data extract. Provider 4 (Wales) appeared to use a future date placeholder (07/07/2037) for open periods of registration.

[1] Based on year and month of birth.

Table S1: Data standards developed from the CPRD Aurum "acceptable patient flag"[1].

CPRD Aurum exclusion criteria	Adapted UK Biobank criteria
Year of birth is empty.	Available for all participants.
Gender other than male, female or indeterminate.	Available for all participants.
Age is greater than 115 at end of follow-up (based on registration end date, death or last collection date).	Same criteria applied.
Patients are not permanently registered.	All participants with registration records were assumed to be permanently registered.
Current registration date is:<br>a) empty.<br>b) greater than the practice's last collection date.<br>c) less than or equal to 01/01/1900.<br>d) equal to or greater than the registration end date.<br>e) prior to the birth year.	The current period of registration was not provided. Individual registration periods were removed if the:<br>a) registration date was missing.<br>b) registration was after the estimated censor date[1].<br>c) registration date was prior to birth.<br>d) registration date was equal to or greater than the deduction date.<br>e) deduction date was prior to birth.
Event dates:<br>a) All recorded health care episodes have empty event dates.<br>b) All recorded health care episodes have invalid events dates (less than or equal to 01/01/1900 or greater than last collection date).<br>c) All recorded health care episodes have dates before the birth year.	Records were not grouped by episode or consultation. Individual clinical event and prescription records were removed if the:<br>a) record date was missing.<br>b) record date was after the estimated date of data extract.<br>c) record date date was prior to birth.

[1] Participants were censored at the earlier of the data extract date (the start of the date range provided by UK Biobank[2]) and the date of death in linked death registry data when cleaning registration record data.

Table S2: Assessment of practice registration record data against quality standards in table S1. Registration date was missing for 3.5% of provider 2 (Scotland) records and registration periods commenced after the data extract for around 2.6% of provider 4 (Wales) records.

Data provider	1 (England Vision)	2 (Scotland)	3 (England TPP)	4 (Wales)	All
**Summary**					
Records	18,530	31,797	246,337	65,149	361,813
Participants	18,508	27,166	164,232	21,145	228,939
Mean number of records	1.0	1.2	1.5	3.1	1.6
**Registration date**					
Missing	5 (0.0%)	1,112 (3.5%)	7 (0.0%)	0 (0.0%)	1,124 (0.3%)
After censoring[1]	14 (0.1%)	1 (0.0%)	87 (0.0%)	1,681 (2.6%)	1,783 (0.5%)
Prior to birth	3 (0.0%)	10 (0.0%)	6 (0.0%)	5 (0.0%)	24 (0.0%)
After deduction	8 (0.0%)	73 (0.2%)	1,709 (0.7%)	94 (0.1%)	1,884 (0.5%)
**Deduction date**					
Prior to birth	0 (0.0%)	0 (0.0%)	1 (0.0%)	0 (0.0%)	1 (0.0%)

[1] Participants were censored at the earlier of the data extract date (the start of the date range provided by UK Biobank[2]) and the date of death in linked death registry data when cleaning registration record data.

Table S3: Assessment of clinical event record data against quality standards in table S1. Date was provided for all provider 4 (Wales) records however 0.1–0.2% of dates were missing for the remaining data providers. All records had a Read v2 or CTV3 code.

Data provider	1 (England Vision)	2 (Scotland)	3 (England TPP)	4 (Wales)	All
**Summary**					
Records	11,972,083	11,365,300	87,483,339	12,835,591	123,656,313
Participants	18,491	27,163	165,179	21,136	230,087
Mean number of records	647.5	418.4	529.6	607.3	537.4
**Record date**					
Missing	22,348 (0.2%)	20,575 (0.2%)	120,281 (0.1%)	0 (0.0%)	163,204 (0.1%)
After data extract[1]	16,294 (0.1%)	1,721 (0.0%)	24,141 (0.0%)	5,180 (0.0%)	47,336 (0.0%)
Prior to birth	135 (0.0%)	150 (0.0%)	1,400 (0.0%)	0 (0.0%)	1,685 (0.0%)
**Record coding**					
No code	0 (0.0%)	0 (0.0%)	0 (0.0%)	0 (0.0%)	0 (0.0%)

[1] Start of the date range provided by UK Biobank[2].

Table S4: Assessment of prescription record data against quality standards in table S1. Dates were largely complete for prescription records. Small numbers of records were recorded after the estimated date of data extract however some of these appear to be repeat prescriptions. Record coding were missing for 1.1% of Scottish records. Drug names were provided in a free–text field for providers 1 to 3 only. UK Biobank note a "system–wide block of missing records prior to 2012"[2] for provider 2 (Scotland) explaining the lower mean number of records.

Data provider	1 (England Vision)	2 (Scotland)	3 (England TPP)	4 (Wales)	All
**Summary**					
Records	6,350,891	4,301,705	39,517,866	7,533,998	57,704,460
Participants	18,299	24,595	160,208	20,488	222,105
Mean number of records	347.1	174.9	246.7	367.7	259.8
**Record date**					
Missing	14 (0.0%)	54 (0.0%)	7,094 (0.0%)	0 (0.0%)	7,162 (0.0%)
After data extract[1]	26,155 (0.4%)	751 (0.0%)	71,623 (0.2%)	17,706 (0.2%)	116,235 (0.2%)
Prior to birth	0 (0.0%)	4 (0.0%)	3 (0.0%)	0 (0.0%)	7 (0.0%)
**Record coding**					
No code	0 (0.0%)	48,968 (1.1%)	0 (0.0%)	0 (0.0%)	48,968 (0.1%)
No description	4 (0.0%)	1 (0.0%)	0 (0.0%)	7,533,998 (100.0%)	7,534,003 (13.1%)
No code or description	0 (0.0%)	1 (0.0%)	0 (0.0%)	0 (0.0%)	1 (0.0%)

[1] Start of the date range provided by UK Biobank[2].

2. IDENTIFYING PERIODS OF EHR DATA COLLECTION

Table S5 is a quantitative description of the challenges presented in UK Biobank registration data including the number of participants with conflicting or overlapping registration periods. A non–negligible proportion of participants from data providers 2 (Scotland) and 3 (TPP data from England practices) have multiple conflicting registration records.

To address the limitations of using practice registration histories to estimate periods of data collection, a rule–based approach was developed to determine the period of EHR data collection for each participant. This is summarised in algorithm A1 and figure S1. An `R` implementation is provided at https://github.com/philipdarke/ukbb-ehr-data.

The algorithm is applied separately to each data provider if a participant has data from multiple providers (for example, a participant that transfers from a medical practice in England to one in Wales). The resultant period(s) of data collection for each data provider are combined and data collection is assumed to be continuous during gaps of less than 1 year.

Table S5: Summary of registration periods for participants with more than one registration record. Providers 2 (Scotland) and 3 (England TPP) have non–negligible proportions of participants with more than one record, with 30.4/76.7% of these periods conflicting respectively. 88.9% of participants in data provider 4 (Wales) have more than one record however almost all periods are consecutive (a new period starts when the previous ends).

Data provider	1 (England Vision)	2 (Scotland)	3 (England TPP)	4 (Wales)	All
**Summary**					
Participants	18,480	26,050	164,197	21,145	227,765
Participants with >1 registration period	16 (0.1%)	3,935 (15.1%)	47,055 (28.7%)	18,792 (88.9%)	70,967 (31.2%)
**Assessment of registration periods**					
Continuous periods[1]	0 (0.0%)	2,113 (53.7%)	9,053 (19.2%)	18,655 (99.3%)	29,887 (42.1%)
Conflicting periods[2]	16 (100.0%)	1,195 (30.4%)	36,112 (76.7%)	5 (0.0%)	38,578 (54.4%)
Gap between periods[3]	0 (0.0%)	936 (23.8%)	13,939 (29.6%)	767 (4.1%)	16,248 (22.9%)

[1] Participants with one or more consecutive registration periods. Consecutive is defined as a registration period starting on the same day, or the day after, the previous registration period ends.
[2] Participants with conflicting registration periods, defined as two or more open registration periods on the same date.
[3] Participants with a gap between registration periods, for example a period of registration ending in 2009 and a subsequent period starting in 2011. The participant may not have been registered with a GP during the interim, or was registered with a GP that has not provided data to UK Biobank.

Algorithm A1: Algorithm used to identify periods of EHR data collection.

	Step	Rationale
1.	Set D_1 to 1 January 1985. Ignore any records before this date.	Periods of data collection are assumed to start no earlier than 1 January 1985[a].
2.	Select the next clinical event record with an attaching observation/test result[b] (or the next prescription record if earlier). Set D_1 to this date.	A prescription record, or an event record with an attaching result, was assumed to indicate potential active data collection.
3.	Check there is an event (i.e. clinical event record *without* an attaching result) in the 31 days previous to D_1. If so, go to step 4. Otherwise go to step 2. Reject participant and end if no dates satisfy this condition[c].	Observations/test results and prescriptions should be accompanied by a recent event record e.g. evidencing a consultation or diagnosis (these records do not have attaching values). The presence of an event record followed by a observation/test result or prescription was assumed to confirm active data collection at date D_1.
4.	If D_1 falls within a period of practice registration, set D_2 to the next deduction date. Otherwise set D_2 to the date of the latest record before the start of the next registration period (or the censor date if earlier). Define period P_1 as the period from D_1 to D_2.	Data collection was assumed to start at date D_1 and continue until the patient de–registered from the practice (if D_1 fell within a period of practice registration) or until the last record before subsequent registration or censoring[d] (if the participant was not registered with a practice at D_1).

[a] GP practices began to adopt EHR systems in the mid–1980s.

[b] A record in `gp_clinical.txt` with a non–empty `value1`, `value2` or `value3` field.

[c] Participant is assumed to have no period of active data collection in the data.

[d] This aims to capture periods of data collection outside of practice registration periods.

Algorithm A1: Algorithm used to identify periods of EHR data collection (continued).

Step	Rationale
5. Identify the periods (if any):<br>a) $P_2, \ldots, P_n$ = subsequent periods of practice registration.<br>b) $G_1, \ldots, G_{n-1}$ = gaps between subsequent registration periods.<br>c) G_n = period from end of final registration period to the censor date.	Participants with multiple periods of practice registration (e.g. as a result of changing GP on relocation) may not be fully captured in the data extract. These participants may feature discontinuous periods of data collection. All additional periods during which EHR data may have been collected are therefore identified.
6. Identify the date of the first and last record within each G_i where $i = 1, ..., n$. Set R_i to the period between the first and last record for each G_i.	The gaps between registration periods are examined to determine whether any records have been recorded outside of periods of practice registration.
7. Active EHR data collection is assumed to take place during:<br>a) P_1.<br>b) $P_2, \ldots, P_n$.<br>c) All periods R_i containing at least one non–prescription record.	Active EHR data collection is assumed to take place:<br>a) During the period from the start of data collection identified in step 4.<br>b) During subsequent periods of practice registration (i.e. it is assumed that a participant did not move from a practice using an EHR system to a paper–based one).<br>c) Between the first and last record during periods where a participant is not registered with a GP practice. Un–registered periods that only contain prescription records are ignored[e].
8. Include gaps between the periods identified in step 7 if they are of length less than 1 year (inclusive).	Participants that move GP practice may not have continuous periods of registration. Gaps of 1 year or less are included to reflect this.

[e] Complete data collection is unlikely to have taken place during un–registered periods that only feature prescription records.

Figure S1: Application of algorithm A1. This synthetic participant corresponds to example 4 in figure 1 in the main manuscript (continued on next page).

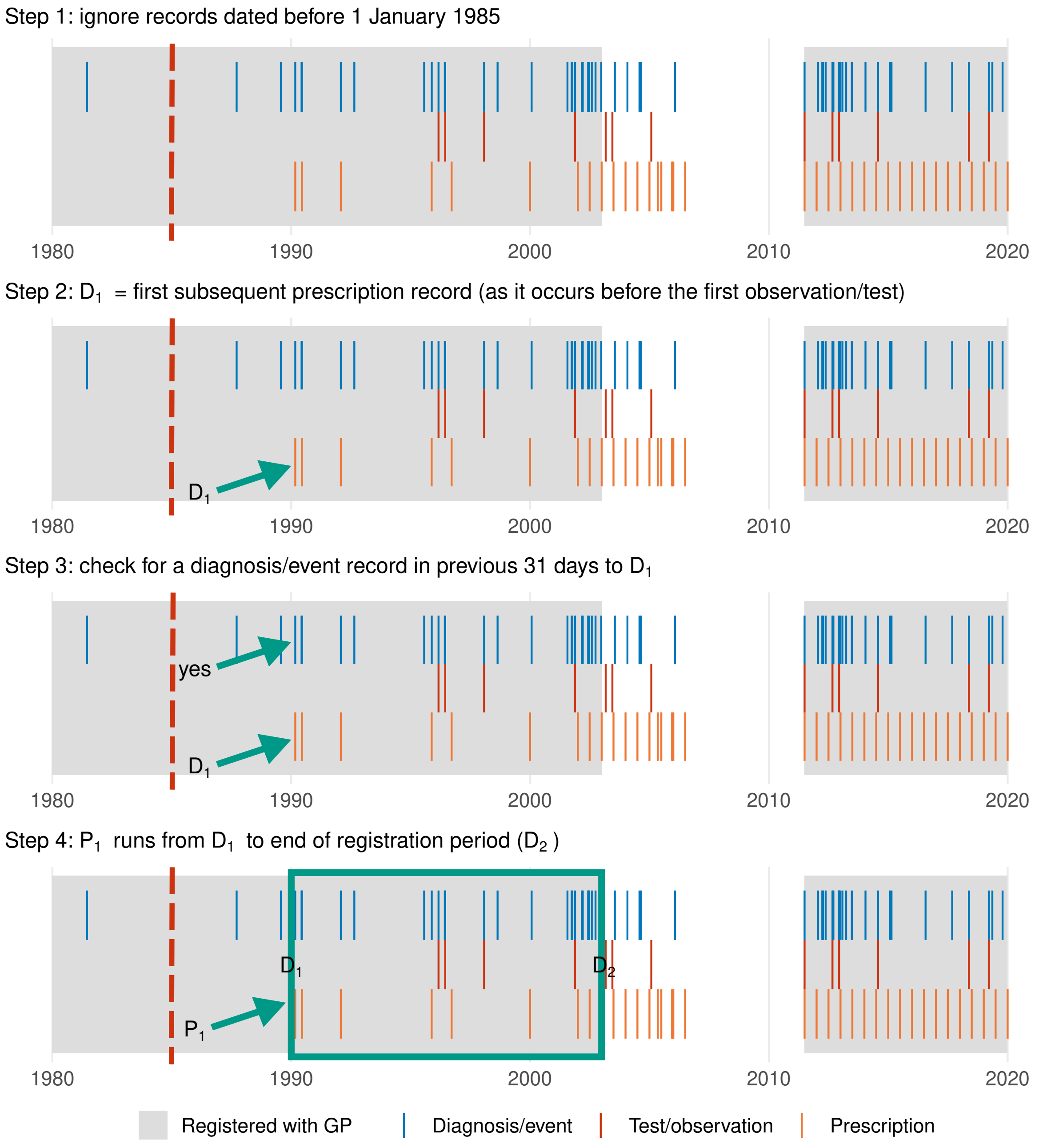

Figure S1: Application of algorithm A1 for a synthetic participant (continued). The boxed areas under step 8 represent the two periods of data collection identified for the participant.

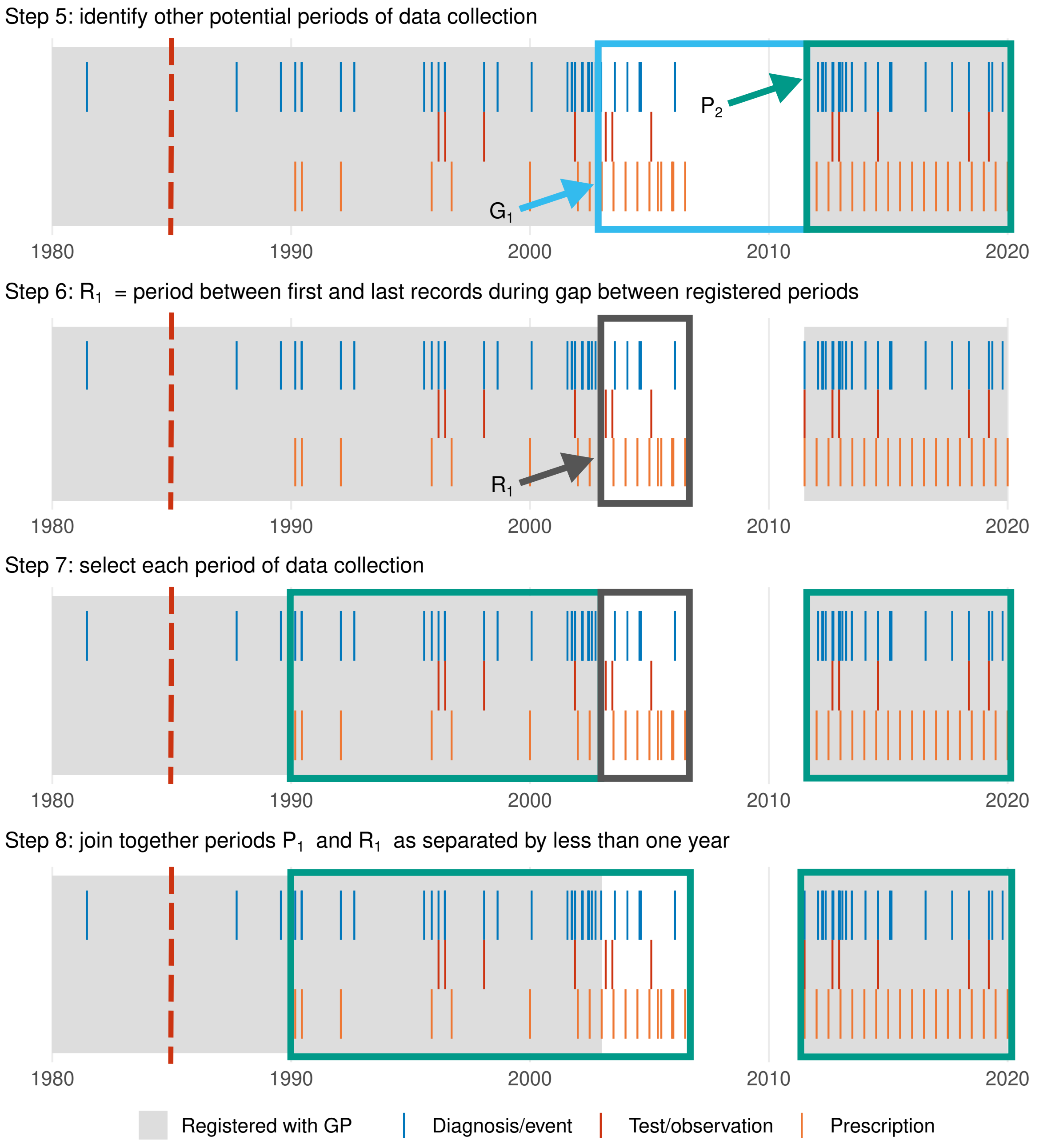

3. EXTRACTING CLINICALLY RELEVANT DATA FROM LINKED EHRS

3.1. Summary of approach

Observations and biomarkers were recorded in up to three value fields. Each data provider adopted a different approach[3] with numeric test results extracted primarily from the first and second value fields. Units, where available, were typically recorded in the `value3` field.

3.2. Unit harmonisation

Neither the units recorded in the `value3` field nor the Read v2/CTV3 code description were a reliable indicator of the units of measurement therefore a rule–based approach was developed to harmonise units of measurement for observations/biomarkers:

a) Candidate units were identified based on the histogram of extracted values (see figure S2), the units recorded in the "value3" field, UK Biobank dictionaries[4] and clinical experience. For example, it was identified that HbA1c values were recorded in both Diabetes Control and Complications Trial (DCCT) and International Federation of Clinical Chemistry (IFCC) units.

b) The range of clinically acceptable values was determined and mapped to each candidate unit for example 4.0–18.6 for DCCT and 20–180 for IFCC units.

c) Values within these ranges were extracted and converted to the harmonised unit shown in table S6. The range with the greatest number of measurements was selected when those identified in step b) overlapped.

d) The median value was taken where multiple test results were recorded on the same day e.g. blood pressure measurements.

e) Measurements and biomarkers recorded at UK Biobank assessment visits were extracted, outliers removed and added to those from the EHR data.

f) The EHR value was discarded when both a UK Biobank and EHR observation were recorded on the same day.

Additional processing was required where multiple measurements were recorded under the same entity type in the Vision practice management system (for example weight in `value1` and BMI in `value2` under code `22A..`).

Figure S2: Histogram showing the bimodal distribution of HbA1c values recorded in the "value1" and "value2" fields. Shaded blue/green areas indicate the range of accepted values for DCCT/IFCC units respectively. Values within each range were extracted and DCCT values converted to IFCC units. Values outside of either range were excluded.

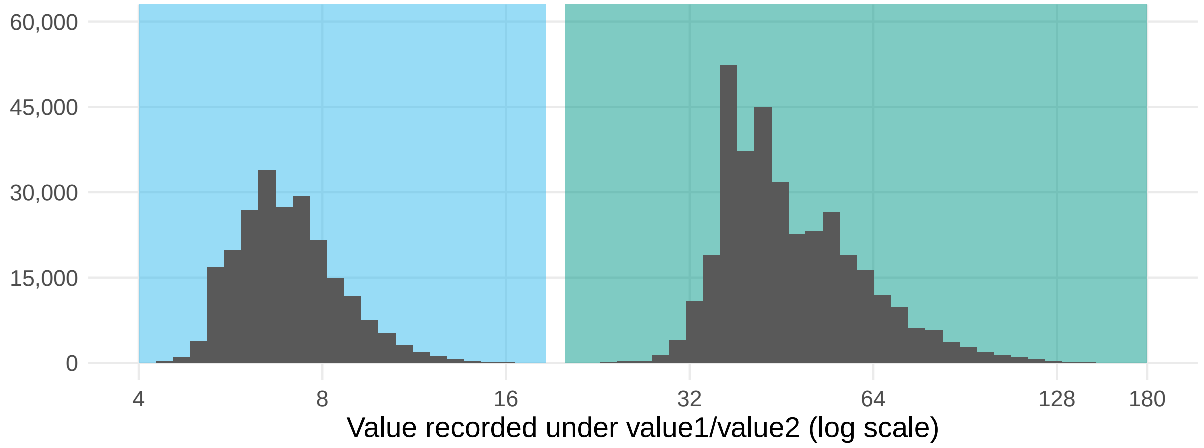

Table S6: Biomarker units and ranges.

Biomarker	Primary units observed in data	Harmonised units	Minimum	Maximum
Glycated hemoglobin	mmol/mol, %	mmol/mol	20	180
Fasting plasma glucose	mmol/L	mmol/L	2	30
Oral glucose tolerance test	mmol/mol	mmol/mol	2	30
Height	m, cm	m	1.1	2.1
Weight	kg	kg	25	200
Body mass index	kg/m^2	kg/m^2	10	80
Waist circumference	cm, inches	cm	50	175

3.3. Prescription data

Table S7 summarises the coding terminology used in UK Biobank. The majority of data is from the TPP practice management system in contrast with the majority of large UK primary care databases (for example The Clinical Practice Research Datalink, The Health Improvement Network and QResearch) which hold data from the Vision and EMIS systems. 75.9% of prescription records have a BNF code and 99.7% of records have a BNF and/or Read v2 code. A pragmatic approach was therefore to develop code sets for these terminologies.

UK Biobank guidance[2] highlights issues including inconsistently formatted BNF codes, missing Read v2 codes and missing drug name data. UK Biobank suggest drug name and quantity data (where available) are used to interpret incomplete/missing code fields.

To handle non–standard coding, primarily BNF codes in TPP data, digit 7 of BNF codes of length 8 was removed for example `02050201` became `0205021` where the final digit is BNF subparagraph. BNF codes of format `00.00.00.00.00`[2] were reformatted for example `02.05.02.01.00` became `0205021`. All Read v2 codes were trimmed to length 5[3].

The majority of prescription records can only be resolved to BNF subparagraph. This is insufficient for some use cases, for example identifying atypical anti–psychotic medication which are included under BNF subparagraph `0402010` along with other anti–psychotic medications. Tables S45 and S46 illustrate how the drug name field can be used to identify drugs where insufficient detail is included in BNF coding.

Table S7: UK Biobank prescription data. `X` indicates the coding terminology used by the data provider.

Code	Country	Data provider	Practice system	Read v2	BNF	dm+d
1	England	Vision	Vision	X	–	X
2	Scotland	Albasoft	EMIS/Vision	X	X	–
3	England	TPP	TPP	–	X	–
4	Wales	SAIL	EMIS/Vision	X	–	–

[2]Note all TPP BNF codes end `00`.
[3]All subsequent characters were `0`.

To determine whether a participant was being prescribed regular medication at a date, prior prescription records were searched for the relevant prescription codes. Figure S3 illustrates the time between prescription records for a selection of drug types. A weekly repeat pattern is present. Previous work based on EMIS data[5] used a 28 day cut–off to determine "active" prescriptions i.e. a prescription within 28 days of the date of interest evidenced a current drug prescription. Based on figure S3, a 90 day cut–off was used for our analysis.

Figure S3: Time between prescriptions in days for a range of drugs. 28 days is the most common interval (except for anti–psychotics) but gaps of 56 days and beyond are common.

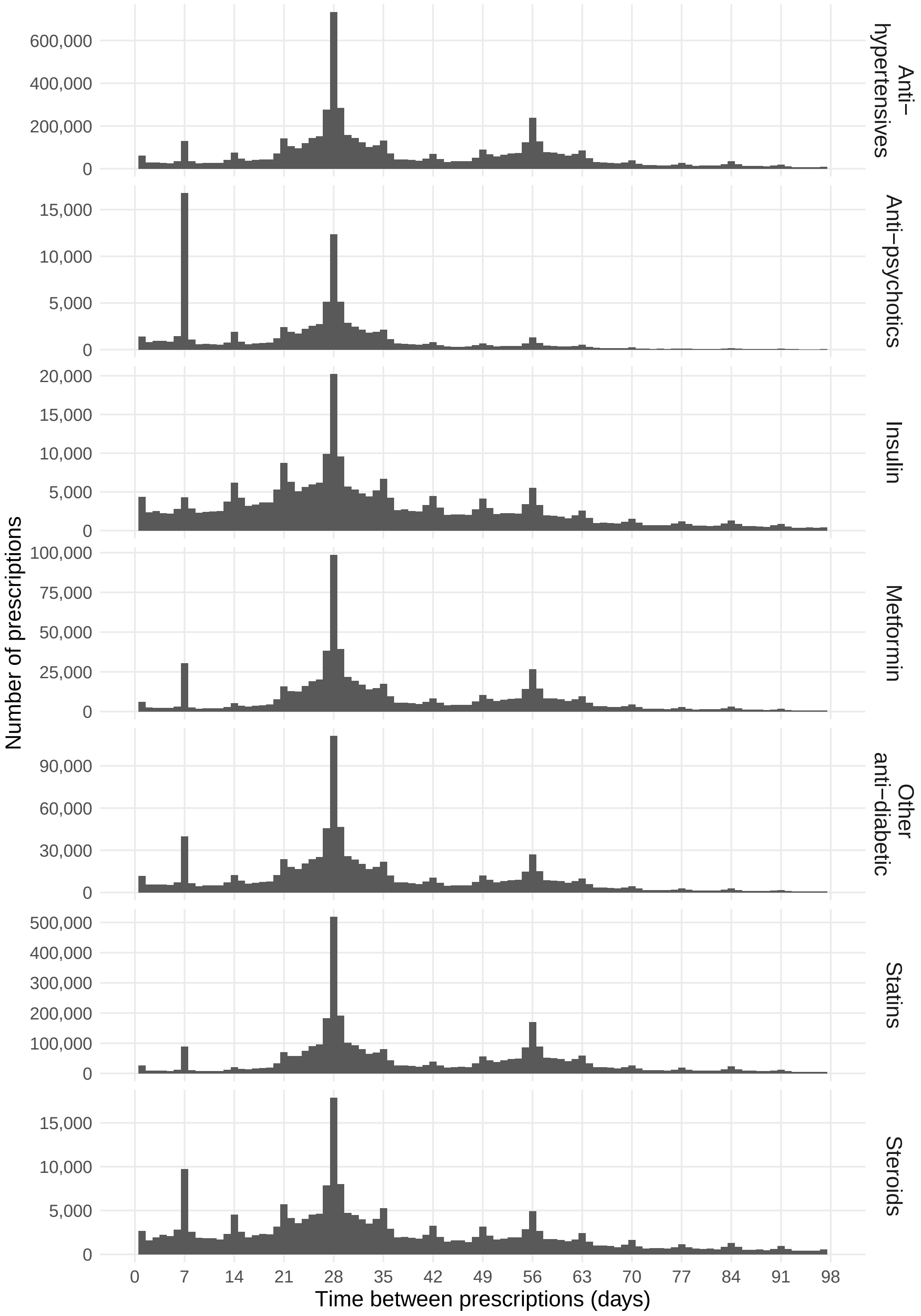

4. VALIDATING THE PROCESSED EHR DATA

Tables S8 to S11 show the agreement between a selection of self–reported conditions and medications and the processed EHR data. Comparison is made as at the first UK Biobank study visit as set out in the main manuscript.

Hypertension, diabetes and myocardial infarction showed high levels of agreement across evaluation metrics. Transient ischaemic attack (TIA) and mental health conditions showed a high number of "false positives" (low precision) where EHR codes were present but participants did not self–report the condition. Potential reasons for this include reluctance to self–report or erroneous code recording (for example a suspected TIA where diagnosis codes are rarely removed if a TIA is later ruled out).

Table S8: Agreement between self–reported and EHR data at the first UK Biobank visit for the conditions and medications used in the QDiabetes–2018 model for **data provider 1 (England Vision)**.

**Active data collection at first UK Biobank visit determined using:**	**Our algorithm (table A1)**			**GP registration records**		
	Sensitivity[1]	Specificity[2]	Precision[3]	Sensitivity[1]	Specificity[2]	Precision[3]
**Presence of a previous diagnostic record:**						
Diabetes	**94.9**	99.8	**94.9**	94.2	99.8	94.7
Hypertension	**71.7**	**98.1**	**93.2**	71.3	98.0	93.0
Myocardial infarction	**68.1**	99.9	93.7	69.5	99.9	93.7
Angina	**49.4**	99.5	74.2	48.2	99.5	**75.7**
Stroke	**57.1**	99.6	**59.9**	55.6	99.6	57.5
Transient ischaemic attack	**58.7**	99.4	**31.9**	58.2	99.4	31.2
Bipolar affective disorder	**81.1**	99.7	35.3	77.8	99.7	**37.3**
Schizophrenia	**94.1**	99.8	**39.0**	93.3	99.8	38.9
Polycystic ovarian syndrome	47.1	99.8	18.6	**50.0**	99.8	**21.1**
**Presence of a prescription record in previous 90 days:**						
Anti–hypertensives	**92.8**	98.8	95.8	91.4	98.8	95.8
Statins	**93.7**	97.6	87.8	92.3	97.6	87.8
Corticosteroids	**55.0**	99.4	50.7	53.0	99.4	**50.8**
Atypical anti–psychotics	**90.2**	100.0	**92.5**	84.2	100.0	91.4

[1] Proportion of self–reporting participants that have a confirmatory EHR record.
[2] Proportion of participants that do not self–report that also do not have an EHR record.
[3] Proportion of participants with an EHR record that also self–report.

Table S9: Agreement between self–reported and EHR data at the first UK Biobank visit for the conditions and medications used in the QDiabetes–2018 model for **data provider 2 (Scotland)**. The impact of missing prescription records[2] results in low sensitivity for this data provider.

**Active data collection at first UK Biobank visit determined using:**	**Our algorithm (table A1)**			**GP registration records**		
	Sensitivity[1]	Specificity[2]	Precision[3]	Sensitivity[1]	Specificity[2]	Precision[3]
**Presence of a previous diagnostic record:**						
Diabetes	**94.6**	99.9	97.7	94.3	99.9	**97.8**
Hypertension	**71.3**	98.5	94.5	70.9	**98.6**	**94.8**
Myocardial infarction	**72.1**	99.8	**91.6**	71.9	99.8	91.5
Angina	55.0	99.4	80.4	**55.5**	99.4	**80.7**
Stroke	60.2	99.5	67.3	**60.4**	**99.6**	**68.2**
Transient ischaemic attack	**61.1**	99.3	**22.6**	59.7	99.3	21.6
Bipolar affective disorder	**71.4**	99.8	**47.9**	70.7	99.8	47.7
Schizophrenia	**90.0**	99.7	29.3	89.7	99.7	**30.2**
Polycystic ovarian syndrome	**61.9**	99.8	**21.7**	60.0	99.8	21.4
**Presence of a prescription record in previous 90 days:**						
Anti–hypertensives	37.3	99.2	93.2	**37.6**	99.2	93.2
Statins	**36.7**	99.3	89.8	37.3	99.3	**89.9**
Corticosteroids	19.5	99.8	51.5	**20.0**	99.8	51.5
Atypical anti–psychotics	31.5	100.0	**89.5**	**33.3**	100.0	88.9

[1] Proportion of self–reporting participants that have a confirmatory EHR record.
[2] Proportion of participants that do not self–report that also do not have an EHR record.
[3] Proportion of participants with an EHR record that also self–report.

Table S10: Agreement between self–reported and EHR data at the first UK Biobank visit for the conditions and medications used in the QDiabetes–2018 model for **data provider 3 (England TPP)**.

**Active data collection at first UK Biobank visit determined using:**	**Our algorithm (table A1)**			**GP registration records**		
	Sensitivity[1]	Specificity[2]	Precision[3]	Sensitivity[1]	Specificity[2]	Precision[3]
**Presence of a previous diagnostic record:**						
Diabetes	94.9	99.8	95.6	94.6	99.8	**95.7**
Hypertension	**72.7**	98.0	93.1	72.5	**98.1**	93.1
Myocardial infarction	71.7	99.9	95.5	71.7	99.9	**95.6**
Angina	**61.7**	99.3	76.4	61.5	99.3	**76.5**
Stroke	**56.0**	99.6	**64.6**	55.2	99.6	64.5
Transient ischaemic attack	55.8	99.4	25.1	55.8	99.4	**25.2**
Bipolar affective disorder	64.8	99.8	42.5	**65.2**	99.8	**42.6**
Schizophrenia	**88.0**	99.8	26.9	87.6	99.8	**27.4**
Polycystic ovarian syndrome	**59.1**	99.8	22.7	58.1	99.8	**22.8**
**Presence of a prescription record in previous 90 days:**						
Anti–hypertensives	92.1	97.9	93.2	**92.2**	**98.0**	**93.3**
Statins	94.1	97.8	89.0	**94.2**	97.8	89.0
Corticosteroids	52.3	99.2	42.9	**52.4**	99.2	42.9
Atypical anti–psychotics	86.6	100.0	86.1	**86.9**	100.0	**86.4**

[1] Proportion of self–reporting participants that have a confirmatory EHR record.
[2] Proportion of participants that do not self–report that also do not have an EHR record.
[3] Proportion of participants with an EHR record that also self–report.

Table S11: Agreement between self–reported and EHR data at the first UK Biobank visit for the conditions and medications used in the QDiabetes–2018 model for **data provider 4 (Wales)**.

**Active data collection at first UK Biobank visit determined using:**	**Our algorithm (table A1)**			**GP registration records**		
	Sensitivity[1]	Specificity[2]	Precision[3]	Sensitivity[1]	Specificity[2]	Precision[3]
**Presence of a previous diagnostic record:**						
Diabetes	**90.1**	99.8	**95.5**	87.6	99.8	95.3
Hypertension	**69.3**	97.9	92.9	67.2	**98.0**	92.9
Myocardial infarction	**60.3**	99.9	93.0	58.2	99.9	**93.4**
Angina	**60.6**	99.2	**72.7**	58.7	**99.3**	72.1
Stroke	48.8	**99.7**	**70.6**	**49.1**	99.6	68.6
Transient ischaemic attack	**45.0**	99.1	10.3	42.9	**99.2**	**11.0**
Bipolar affective disorder	**72.7**	99.7	31.6	70.6	99.7	**34.3**
Schizophrenia	73.7	99.8	31.8	73.7	**99.9**	**35.0**
Polycystic ovarian syndrome	43.8	99.9	25.0	**50.0**	99.9	**28.6**
**Presence of a prescription record in previous 90 days:**						
Anti–hypertensives	**92.8**	98.5	**95.0**	88.9	**98.6**	94.9
Statins	**94.1**	97.9	89.9	**90.1**	**98.1**	89.9
Corticosteroids	59.7	99.3	54.9	**58.9**	99.3	**55.8**
Atypical anti–psychotics	**72.7**	99.9	**75.5**	70.9	99.9	75.0

[1] Proportion of self–reporting participants that have a confirmatory EHR record.
[2] Proportion of participants that do not self–report that also do not have an EHR record.
[3] Proportion of participants with an EHR record that also self–report.

5. LONGITUDINAL DIABETES PHENOTYPING

GP diagnosis codes were used to identify disease sub–type, and the prescription of anti–diabetic medication and repeated measures of blood tests (including those rarely collected by UK Biobank such as fasting blood glucose) identified related health states including non–diabetic hyperglycaemia and remission.

5.1. Code set development

To identify diabetes diagnoses, all Read v2 codes starting `C10` were labelled as corresponding to type 1 diabetes, type 2 diabetes, a known other diabetes type or unknown diabetes type (for example the generic diabetes code `C10...`). Codes that did not correspond to a diabetes diagnosis were excluded. Equivalent CTV3 codes were mapped using the NHS Digital Technology Reference data Update Distribution[6]. Code descriptions were searched for the terms "diabetes" and "diabetic" to identify additional candidate codes including those not identified by the forward mapping of Read v2 codes.

A similar approach was used to develop code sets for evidence of the prescription of clinically relevant biomarkers including blood glucose test results. Prescriptions were identified based on relevant Read v2 and BNF chapters. Code sets were reviewed by two authors with diabetes expertise and codes included only where there was full agreement. Code sets are summarised in section 7.

5.2. Diabetes incidence criteria

Participants were assumed to enter a diabetic state at the date of the first diagnosis code. Where multiple diabetes sub–types were present in the data, participants were assumed to have type 2 diabetes with the exception of those under age 35 *and* with an insulin prescription prior to one year after diagnosis who were assumed to have type 1 diabetes.

5.3. Diabetes remission criteria

Remission was defined as the cessation of all diabetes medication followed by two sub–diabetic blood glucose test results (glycated hemoglobin (HbA1c) $<$ 48 mmol/mol or fasting plasma glucose $<$ 7.0 mmol/l) separated by at least six months[7].

5.4. Pre–diabetes criteria

Normoglycaemic participants were deemed to enter a pre–diabetic state on the first date they met National Institute for Health and Clinical Excellence PH38 criteria[8]:

a) HbA1c $\geq$ 42 mmol/mol or fasting plasma glucose $\geq$ 5.5 mmol/l (two–hour oral glucose tolerance test results $\geq$ 7.8 mmol/mol were also included to capture those with impaired glucose tolerance[9])

b) no previous diabetes diagnosis

c) no diagnosis in the subsequent three months (excluding gestational diabetes) to allow for the delayed recording of a clinical code following a diagnosis based on a blood glucose test.

Glucose tests during periods of gestational diabetes or anti–diabetes medication were not tested against these criteria.

6. PERFORMANCE OF NHS–APPROVED DIABETES RISK PREDICTION TOOLS

6.1. QDiabetes–2018

QDiabetes–2018 was evaluated in line with Hippisley-Cox and Coupland [5] using a study start of 1 January 2005 and the same inclusion criteria. Performance was evaluated using our algorithm to identify periods of data collection and our phenotyping approach to determine predictors and outcomes (tables S12 and S14) and, for comparison, assuming complete data collection during periods of GP registration record and predictors/outcomes determined as in Hippisley-Cox and Coupland [5] (tables S13 and S15).

Our algorithm results in a broadly the same number of eligible participants (205,901 *vs* 205,290) but longer post visit follow–up (mean of 10.6 *vs* 9.7 years).

Table S12: Population used to assess QDiabetes–2018 performance. Periods of data collection identified using our algorithm and predictors/outcomes using our phenotyping approach.

	Study population	Excluded[1]
**Summary**		
Participants	205,901	8,151
Male	44.8%	62.1%
Mean age (years)	53.7	57.7
Mean Townsend score	-1.4	-0.4
Mean body mass index (kg/m^2)	26.1	30.5
Complete data[2]	79.2%	91.4%
$\geq$ 1 prescription record[3]	85.1%	91.0%
Follow–up (years)	10.6	9.7
**Blood glucose**		
Mean fasting plasma glucose (mmol/L)	5.1	8.5
Mean HbA1c (mmol/mol)	35.3	58.7
**Ethnicity**		
White	95.2%	86.8%
**Smoking status**		
Non–smoker	57.5%	51.2%
Former smoker	21.0%	32.6%
Light smoker	1.1%	0.8%
Moderate smoker	10.9%	11.7%
Heavy smoker	0.7%	0.7%
**Medical characteristics**		
Family history of diabetes	26.1%	51.8%
Treated hypertension	12.1%	48.0%
Cardiovascular disease	4.6%	19.1%
Schizophrenia/bipolar affective disorder	0.7%	1.2%
Learning disability	0.1%	0.3%
Gestational diabetes[4]	0.1%	1.0%
Polycystic ovary syndrome[4]	0.6%	1.3%
**Current drugs**		
Statins	6.9%	54.7%
Atypical antipsychotics	0.2%	0.5%
Corticosteroids	1.0%	2.3%

[1] Participants excluded from evaluation (primarily due to pre–existing diabetes).
[2] Participants with sex, age, ethnicity, Townsend score, BMI and smoking status.
[3] At least one prescription record on/prior to study entry.
[4] As proportion of female participants.

Table S13: Population used to assess QDiabetes–2018 performance. Periods of data collection identified using GP registration records and predictors/outcomes as in Hippisley-Cox and Coupland [5].

	Study population	Excluded[1]
**Summary**		
Participants	205,290	6,341
Male	45.0%	60.8%
Mean age (years)	53.9	57.6
Mean Townsend score	-1.4	-0.4
Mean body mass index (kg/m^2)	26.1	30.5
Complete data[2]	78.8%	91.8%
≥ 1 prescription record[3]	84.8%	92.4%
Follow–up (years)	9.7	8.7
**Blood glucose**		
Mean fasting plasma glucose (mmol/L)	5.1	9.0
Mean HbA1c (mmol/mol)	35.9	62.7
**Ethnicity**		
White	95.2%	86.8%
**Smoking status**		
Non–smoker	56.7%	52.5%
Former smoker	21.1%	32.3%
Light smoker	1.1%	0.7%
Moderate smoker	10.6%	11.9%
Heavy smoker	0.7%	0.7%
**Medical characteristics**		
Family history of diabetes	26.2%	52.6%
Treated hypertension	10.1%	42.5%
Cardiovascular disease	3.5%	14.6%
Schizophrenia/bipolar affective disorder	0.6%	1.0%
Learning disability	0.1%	0.2%
Gestational diabetes[4]	0.1%	0.8%
Polycystic ovary syndrome[4]	0.5%	1.1%
**Current drugs**		
Statins	5.2%	39.4%
Atypical antipsychotics	0.2%	0.4%
Corticosteroids	0.6%	1.3%

[1] Participants excluded from evaluation (primarily due to pre–existing diabetes).
[2] Participants with sex, age, ethnicity, Townsend score, BMI and smoking status.
[3] At least one prescription record on/prior to study entry.
[4] As proportion of female participants.

Table S14: Performance of QDiabetes–2018 model by data provider. Periods of data collection identified using our algorithm and predictors/outcomes using our phenotyping approach. Validation results on QResearch data[5] are shown for comparison (*QDiabetes*).

	**Model A**		**Model B**		**Model C**	
	Male	Female	Male	Female	Male	Female
**Number of participants**						
1 (England Vision)	5,982	8,144	682	689	891	1,021
2 (Scotland)	7,499	10,191	265	247	1,182	1,133
3 (England TPP)	50,824	66,631	5,376	5,626	6,607	7,598
4 (Wales)	6,212	8,306	1,308	1,430	765	766
All providers	70,255	92,902	7,594	7,941	9,222	10,212
**Concordance index**[a]						
1 (England Vision)	0.790	0.811	0.836	0.817	0.816	0.902
2 (Scotland)	0.804	0.830	0.880	0.867	0.954	0.903
3 (England TPP)	0.782	0.834	0.832	0.859	0.886	0.904
4 (Wales)	0.762	0.817	0.798	0.883	0.804	0.888
All providers	0.781	0.832	0.831	0.877	0.882	0.904
QDiabetes	*0.814*	*0.834*	*0.866*	*0.899*	*0.855*	*0.878*
**R–squared**[b]						
1 (England Vision)	0.372	0.478	0.504	0.507	0.500	0.487
2 (Scotland)	0.402	0.466	0.571	0.680	0.612	0.651
3 (England TPP)	0.381	0.485	0.470	0.564	0.519	0.544
4 (Wales)	0.356	0.452	0.402	0.570	0.482	0.627
All providers	0.380	0.480	0.465	0.563	0.519	0.556
QDiabetes	*0.466*	*0.505*	*0.584*	*0.633*	*0.555*	*0.603*
**D–statistic**[c]						
1 (England Vision)	1.575	1.958	2.062	2.077	2.049	1.993
2 (Scotland)	1.677	1.913	2.360	2.981	2.571	2.798
3 (England TPP)	1.606	1.987	1.928	2.327	2.125	2.234
4 (Wales)	1.522	1.861	1.679	2.357	1.973	2.651
All providers	1.603	1.964	1.908	2.325	2.125	2.288
QDiabetes	*1.91*	*2.07*	*2.42*	*2.69*	*2.28*	*2.52*

[a] Harrell's C.

[b] Proportion of variance in the time–to–event explained by QDiabetes–2018.

[c] Royston's D–statistic.

Table S15: Performance of QDiabetes–2018 model by data provider. Periods of data collection identified using GP registration records and predictors/outcomes as in Hippisley-Cox and Coupland [5]. Validation results on QResearch data[5] are shown for comparison (*QDiabetes*).

	**Model A**		**Model B**		**Model C**	
	Male	Female	Male	Female	Male	Female
**Number of participants**						
1 (England Vision)	6,041	8,173	948	1,042	1,482	1,802
2 (Scotland)	7,103	10,123	266	268	1,115	1,310
3 (England TPP)	50,304	66,340	5,883	6,202	7,784	8,795
4 (Wales)	6,010	8,077	1,258	1,383	584	560
All providers	69,249	92,479	8,340	8,882	10,828	12,311
**Concordance index[a]**						
1 (England Vision)	0.787	0.802	0.889	0.882	0.828	0.900
2 (Scotland)	0.793	0.825	0.856	0.809	0.892	0.920
3 (England TPP)	0.785	0.824	0.826	0.863	0.886	0.906
4 (Wales)	0.756	0.814	0.807	0.890	0.769	0.805
All providers	0.784	0.824	0.837	0.883	0.877	0.903
QDiabetes	*0.814*	*0.834*	*0.866*	*0.899*	*0.855*	*0.878*
**R–squared[b]**						
1 (England Vision)	0.377	0.465	0.533	0.545	0.439	0.529
2 (Scotland)	0.391	0.453	0.560	0.516	0.452	0.529
3 (England TPP)	0.390	0.480	0.474	0.553	0.478	0.574
4 (Wales)	0.376	0.476	0.413	0.641	0.333	0.580
All providers	0.388	0.476	0.480	0.562	0.464	0.572
QDiabetes	*0.466*	*0.505*	*0.584*	*0.633*	*0.555*	*0.603*
**D–statistic[c]**						
1 (England Vision)	1.594	1.909	2.185	2.242	1.812	2.406
2 (Scotland)	1.639	1.861	2.311	2.115	1.860	2.169
3 (England TPP)	1.637	1.966	1.945	2.275	1.959	2.377
4 (Wales)	1.587	1.951	1.718	2.732	1.446	2.405
All providers	1.631	1.949	1.965	2.319	1.905	2.364
QDiabetes	*1.91*	*2.07*	*2.42*	*2.69*	*2.28*	*2.52*

[a] Harrell's C.
[b] Proportion of variance in the time–to–event explained by QDiabetes–2018.
[c] Royston's D–statistic.

Figure S4: Calibration of QDiabetes–2018 model on UK Biobank data (all data providers). Periods of data collection identified using our algorithm and outcomes using our phenotyping approach.

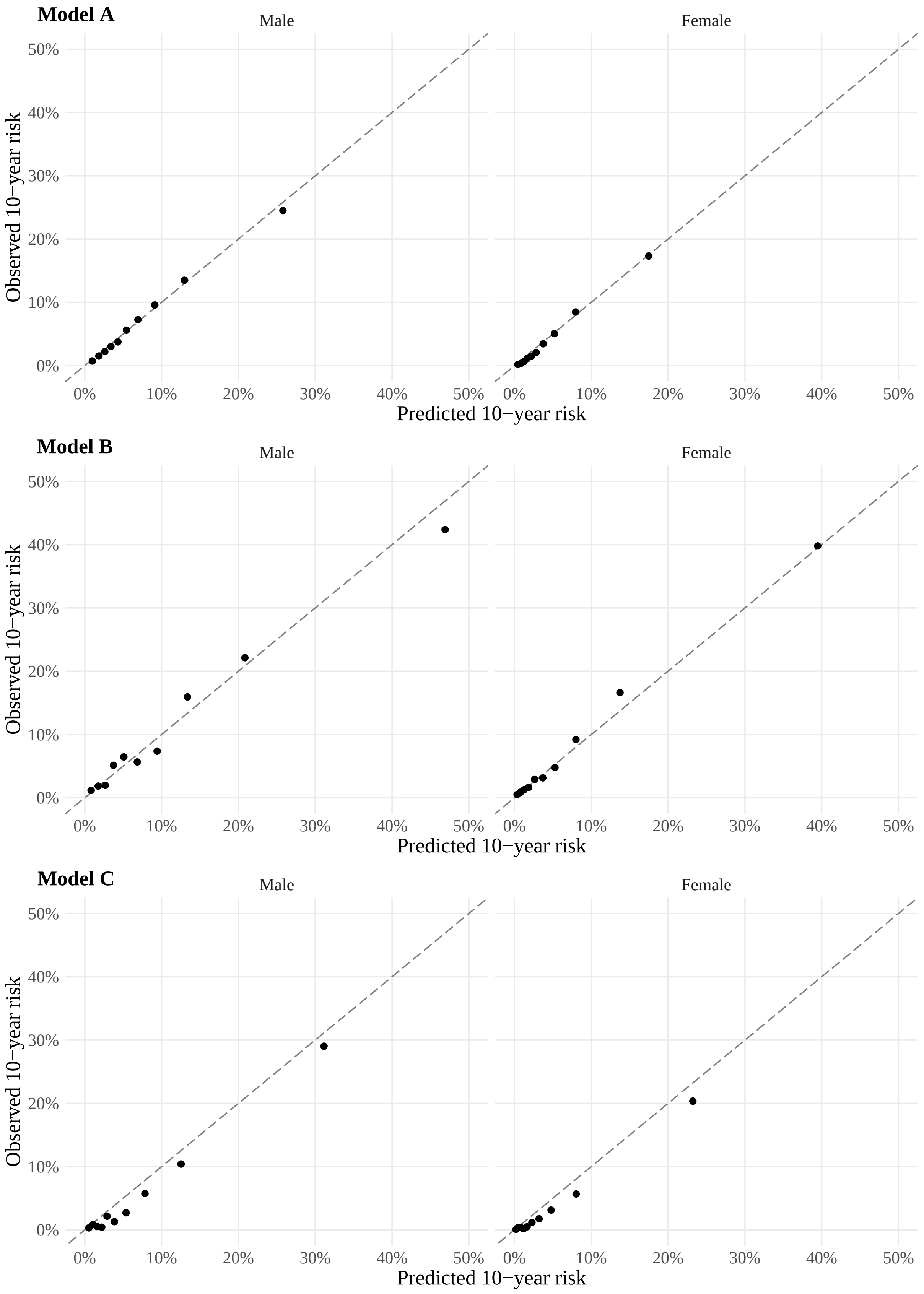

Figure S5: Calibration of QDiabetes–2018 model on UK Biobank data (all data providers). Periods of data collection identified using GP registration records and outcomes as in Hippisley-Cox and Coupland [5].

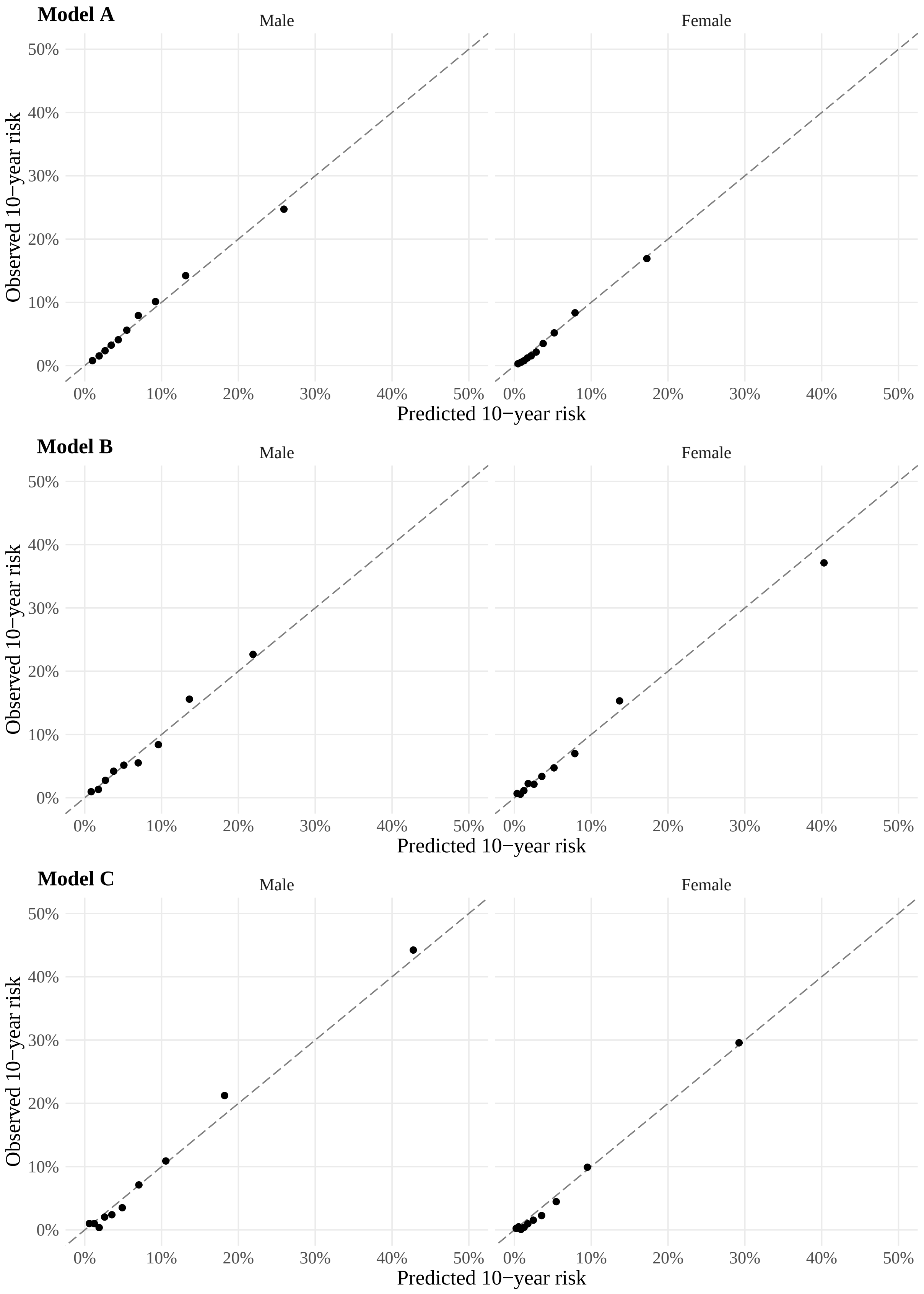

6.2. Leicester risk score

The Leicester risk score was calculated as in Gray et al. [10]. Performance was evaluated when predicting the 5 year incidence of diabetes at the first UK Biobank visit. 5 year performance was used due to the relatively small number of UK Biobank participants with 10 years of EHR follow–up following the first study visit. Our algorithm was used to identify periods of data collection and our phenotyping approach to determine outcomes (table S16). For comparison, the evaluation was also carried out assuming complete data collection during periods of GP registration record and outcomes determined as in Hippisley-Cox and Coupland [5] (table S17). Results are shown in table S18.

Our algorithm results in a larger number of participants deemed to have EHR data collection from the first UK Biobank study visit (186,208 *vs* 181,233 when using registration record data) and longer post visit follow–up (mean of 7.4 *vs* 6.9 years). Leicester score performance for the 5 year incidence of diabetes was generally higher using our algorithm and phenotyping approach.

Table S16: Population used to assess Leicester Score performance (all data providers using algorithm A1 to determine periods of data collection).

	Study population	Excluded[1]
**Summary**		
Participants	186,208	10,542
Male	44.4%	61.1%
Mean age (years)	57.0	60.1
Complete data[2]	99.3%	98.7%
Follow–up (years)	7.4	7.2
**Ethnicity**		
White	95.5%	88.2%
**Medical characteristics**		
Mean body mass index (kg/m^2)	27.3	31.5
Mean waist circumference (cm)	89.6	102.9
Family history of diabetes	25.6%	51.0%
Treated hypertension	35.6%	79.8%

[1] Participants excluded from evaluation (primarily due to pre–existing diabetes).
[2] Participants with sex, age, ethnicity, waist circumference and BMI.

Table S17: Population used to assess Leicester Score performance (all data providers assuming data collection during periods of GP registration).

	Study population	Excluded[1]
**Summary**		
Participants	181,233	10,273
Male	44.6%	61.3%
Mean age (years)	57.0	60.2
Complete data[2]	99.3%	98.7%
Follow–up (years)	6.9	6.8
**Ethnicity**		
White	95.6%	88.4%
**Medical characteristics**		
Mean body mass index (kg/m^2)	27.3	31.5
Mean waist circumference (cm)	89.6	102.9
Family history of diabetes	25.5%	51.0%
Treated hypertension	35.6%	79.8%

[1] Participants excluded from evaluation (primarily due to pre–existing diabetes).
[2] Participants with sex, age, ethnicity, waist circumference and BMI.

Table S18: Confusion matrices for the 5 year incidence of diabetes following the first UK Biobank visit using the Leicester score. For comparison, Barber et al. [11] reported sensitivity of 89.2% and specificity of 42.3% for 10 year diabetes prediction.

**Active data collection/ diabetes outcomes determined using:**	**Our algorithm/phenotyping tool**			**GP registration records/as QDiabetes[5]**		
	Score < 16	Score ≥ 16		Score < 16	Score ≥ 16	
**Provider 1 (England Vision)**						
– Diabetes diagnosis	110	555	**sensitivity 83.5%**	124	529	sensitivity 81.0%
– No diabetes diagnosis	8,832	5,893	**specificity 60.0%**	7,928	5,389	specificity 59.5%
**Provider 2 (Scotland)**						
– Diabetes diagnosis	150	712	**sensitivity 82.6%**	160	686	sensitivity 81.1%
– No diabetes diagnosis	12,866	7,045	specificity 64.6%	12,381	6,766	**specificity 64.7%**
**Provider 3 (England TPP)**						
– Diabetes diagnosis	653	4,110	**sensitivity 86.3%**	744	4,198	sensitivity 84.9%
– No diabetes diagnosis	68,899	51,213	**specificity 57.4%**	63,882	47,575	specificity 57.3%
**Provider 4 (Wales)**						
– Diabetes diagnosis	113	725	**sensitivity 86.5%**	80	510	sensitivity 86.4%
– No diabetes diagnosis	9,209	6,575	specificity 58.3%	5,972	4,259	**specificity 58.4%**
**All data providers**						
– Diabetes diagnosis	1,028	6,108	**sensitivity 85.6%**	1,101	5,901	sensitivity 84.3%
– No diabetes diagnosis	99,901	70,788	specificity 58.5%	89,589	72,175	specificity 58.5%

7. EHR CODE SETS

7.1. Diabetes diagnosis and related coding

Table S19: Type 1 diabetes diagnosis.

Read v2	CTV3	Description	Records
	X40J4	Type I diabetes mellitus	4,719
C10E.		Type 1 diabetes mellitus	720
C108.		Insulin dependent diabetes mellitus	295

Table S20: Type 2 diabetes diagnosis.

Read v2	CTV3	Description	Records
	X40J5	Type II diabetes mellitus	56,358
C10F.		Type 2 diabetes mellitus	19,981
C10FJ	X40J6	Insulin treated Type 2 diabetes mellitus	1,867
C109.		Non-insulin dependent diabetes mellitus	1,812
C109J	X40J6	Insulin treated Type 2 diabetes mellitus	1,580
	XaELQ	Type II diabetes mellitus without complica...	303
	C1097	Type II diabetes mellitus - poor control	243
	C1001	Diabetes mellitus: [adult onset, with no m...	192
	XaIzR	Type II diabetes mellitus with persistent ...	188
	X40JE	Metabolic syndrome X	122

Table S21: Diabetes diagnosis (unknown type).

Read v2	CTV3	Description	Records
C10..	C10..	Diabetes mellitus	19,051
	X40Jc	Poor glycaemic control	102

Table S22: Gestational diabetes diagnosis codes.

Read v2	CTV3	Description	Records
L1809	L1808	Gestational diabetes mellitus	257

Table S23: Family history of diabetes codes.

Read v2	CTV3	Description	Records
1252.	1252.	FH: Diabetes mellitus	39,231
ZV180	ZV180	[V]Family history of diabetes mellitus	3,563
1253.	XaKYz	FH: Diabetes mellitus in first degree rela...	3,470
ZV198	ZV180	[V]Family history of diabetes mellitus	3,410
	XaVw4	Family history of diabetes mellitus type II	1,095
12520	XaVw3	Family history of diabetes mellitus type 1	517

7.2. Hypertension diagnosis

Table S24: Hypertension diagnosis codes.

Read v2	CTV3	Description	Records
G20..	XE0Uc	Essential hypertension	175,195
G2...	G2...	Hypertensive disease	47,097
	XE0Ub	Hypertension	15,894
G20z.	XE0Ud	Essential hypertension NOS	7,906
	XM02V	Raised blood pressure	2,705
G2z..	G2z..	Hypertensive disease NOS	2,152
G201.	G201.	Benign essential hypertension	1,887
	XM09K	Raised blood pressure reading	927
G25..	XaZWm	Stage 1 hypertension (NICE - National Inst...	403
G202.	G202.	Systolic hypertension	321

7.3. Cardiovascular disease diagnosis

Table S25: Myocardial infarction diagnosis codes.

Read v2	CTV3	Description	Records
G30..	XE0Uh	Acute myocardial infarction	8,290
G3071	XaIwY	Acute non-ST segment elevation myocardial ...	2,137
	X200E	Myocardial infarction	1,007
G30X0	XaIwM	Acute ST segment elevation myocardial infa...	923
G30z.	G30z.	Acute myocardial infarction NOS	618
G308.	G308.	Inferior myocardial infarction NOS	465
G301z	G301z	Anterior myocardial infarction NOS	189

Table S26: Angina diagnosis codes.

Read v2	CTV3	Description	Records
	G33..	Angina	9,776
662K0	662K0	Angina control - good	7,410
G33..		Angina pectoris	4,207
662K.	662K.	Angina control	1,825
G33z7	X2008	Stable angina	852
G33zz	G33z.	Angina pectoris NOS	523
G33z.	G33z.	Angina pectoris NOS	502
662K3	662K3	Angina control - worsening	331
662K1	662K1	Angina control - poor	219
662K2	662K2	Angina control - improving	192

Table S27: Stroke diagnosis codes.

Read v2	CTV3	Description	Records
G66..	XE2aB	Stroke and cerebrovascular accident unspec...	3,489
G60..	Xa1uW	Subarachnoid haemorrhage	1,034
	X00D1	Cerebrovascular accident	995
G64z.	XE0VJ	Cerebral infarction NOS	641
G64..	XE0VI	Cerebral arterial occlusion	565
	G66..	CVA - cerebrovascular accident (& unspecif...	346
G634.	G634.	Carotid artery stenosis	329
	Xa0kZ	Cerebral infarction	326
G61..		Intracerebral haemorrhage	223
	XE0VF	Cerebral parenchymal haemorrhage	170

Table S28: Transient ischaemic attack diagnosis codes.

Read v2	CTV3	Description	Records
G65..		Transient cerebral ischaemia	3,476
	XE0VK	Transient ischaemic attack	3,444
G65zz	G65z.	Transient cerebral ischaemia NOS	1,255
G65z.	G65z.	Transient cerebral ischaemia NOS	1,102

7.4. Other conditions

Table S29: Learning disability diagnosis codes.

Read v2	CTV3	Description	Records
	E....	Mental health disorder	492
918e.	XaKYb	On learning disability register	153
13Z4E	13Z4E	Learning difficulties	111

Table S30: Bipolar affective disorder diagnosis codes.

Read v2	CTV3	Description	Records
	X00SM	Bipolar disorder	974
Eu31z	Eu31z	[X]Bipolar affective disorder, unspecified	648
Eu31.		[X]Bipolar affective disorder	366
	X00SL	Hypomania	287
E116.	E116.	Mixed bipolar affective disorder	246
Eu317	Eu317	[X]Bipolar affective disorder, currently i...	227
E11z1	E11z1	Rebound mood swings	198
E117.	E117.	Unspecified bipolar affective disorder	125
E116z	E116z	Mixed bipolar affective disorder, NOS	115
	E11..	Affective psychoses (& [bipolar] or [depre...	106

Table S31: Schizophrenia diagnosis codes.

Read v2	CTV3	Description	Records
E10..	E10..	Schizophrenic disorders	568
E103.	E103.	Paranoid schizophrenia	526
	Eu20.	Schizophrenia	384
E10z.	E10z.	Schizophrenia NOS	296
	E12..	Paranoid disorder	157
E12z.	E12z.	Paranoid psychosis NOS	121
	Eu25.	Schizoaffective disorder	112
1BH3.	XaKUl	Paranoid ideation	101

Table S32: Polycystic ovarian syndrome diagnosis codes.

Read v2	CTV3	Description	Records
C164.	XE101	Polycystic ovaries	668
	X406n	Polycystic ovary syndrome	223

7.5. Anthropometric

Body Mass Index (BMI) was extracted using the codes in table S33. BMI was calculated from height (table S34) and weight (table S35) when weight was recorded without an accompanying BMI. The previous height measurement was carried forward if a new height was not recorded alongside the new weight. Units were harmonised to m, kg and kg/m^2 and outliers dropped.

Table S33: Body mass index codes.

Read v2	CTV3	Description	Records
	22K..	Body mass index - observation	1,081,253
22K..		Body Mass Index	434,061
22K5.	22K5.	Body mass index 30+ - obesity	20,806
22K4.	22K4.	Body mass index index 25-29 - overweight	16,458
	22K1.	Body mass index normal K/M2	16,232
22K2.		Body Mass Index high K/M2	11,719
	22K2.	Body mass index high K/M2	9,574
22K1.		Body Mass Index normal K/M2	5,794
22K8.	XaJqk	Body mass index 20-24 - normal	5,384
22K7.	XaJJH	Body mass index 40+ - severely obese	1,985

Table S34: Height codes.

Read v2	CTV3	Description	Records
229..	229..	O/E - height	996,507
22Z..	22Z..	Height and Weight	1,083
229Z.	229Z.	O/E - height NOS	952
2293.	2293.	O/E -height within 10% average	438
2296.	2296.	O/E - loss of height	114

Table S35: Weight codes.

Read v2	CTV3	Description	Records
22A..	22A..	O/E - weight	1,645,908
22AZ.	22AZ.	O/E - weight NOS	2,938
22A4.	XE1h3	O/E - weight 10-20% over ideal	2,287
22A3.	22A3.	O/E - weight within 10% ideal	2,276
	XM01G	Weight observation	1,875
22Z..	22Z..	Height and Weight	1,083
222A.	222A.	O/E - obese	702
	XM1YD	O/E - overweight	613
22A5.		O/E - weight >20% over ideal	606
	X76C9	Weight gain	525

Table S36: Waist circumference codes.

Read v2	CTV3	Description	Records
22N0.	Xa041	Waist circumference	110,068

7.6. Smoking status

See codes and mapping in table S49.

Table S37: Smoking status codes and mapping. Table shows the 10 most prevalent codes with at least 1,000 records for each QDiabetes category.

Read v2	CTV3	Description	Level	Records
**Non–smoker = never smoked/current non–smoker**				
1371.	XE0oh	Never smoked tobacco		935,159
137L.	137L.	Current non-smoker		96,407
	Ub0oq	Non-smoker		52,527
13WK.	XaIn9	No smokers in the household		2,616
	1371.	Non-smoker (& [never smoked tobacco])		1,975
**Former smoker**				
	Ub1na	Ex-smoker		327,389
137S.		Ex smoker		152,125
137j.	Xa1bv	Ex-cigarette smoker		37,301
137K.	137K.	Stopped smoking		34,400
1379.	1379.	Ex-moderate smoker (10-19/day)	Moderate	24,366
137T.	137T.	Date ceased smoking		15,743
1378.	1378.	Ex-light smoker (1-9/day)	Light	14,900
137F.	137F.	Ex-smoker - amount unknown		14,362
137A.	137A.	Ex-heavy smoker (20-39/day)	Heavy	13,115
1377.	1377.	Ex-trivial smoker (<1/day)	Trivial	5,740
**Light smoker = current trivial/light smoker**				
	1373.	Light cigarette smoker (1-9 cigs/day)	Light	6,686
1373.		Light smoker - 1-9 cigs/day	Light	3,474
1372.		Trivial smoker - <1 cig/day	Trivial	1,515
	XE0oi	Trivial cigarette smoker (less than one ci...	Trivial	1,387
**Moderate smoker = current moderate smoker/smoking level unknown**				
	137R.	Smoker		96,114
137P.	XE0oq	Cigarette smoker		88,118
137R.		Current smoker		23,324
	1374.	Moderate cigarette smoker (10-19 cigs/day)	Moderate	9,571
137G.	137G.	Trying to give up smoking		8,303
1374.		Moderate smoker - 10-19 cigs/d	Moderate	5,873
137J.	137J.	Cigar smoker		4,402
137d.	XaIkY	Not interested in stopping smoking		3,710
137c.	XaIkW	Thinking about stopping smoking		3,254
137M.	137M.	Rolls own cigarettes		2,761
**Heavy smoker = current heavy/very heavy smoker**				
	1375.	Heavy cigarette smoker (20-39 cigs/day)	Heavy	5,101
1375.		Heavy smoker - 20-39 cigs/day	Heavy	3,714

7.7. Blood glucose

Table S38: Fasting plasma glucose codes.

Read v2	CTV3	Description	Records
44g1.	44g1.	Plasma fasting glucose level	234,630
44TK.	XE2mq	Fasting blood glucose level	75,106
44f1.	44f1.	Serum fasting glucose level	59,163
44T2.		Fasting blood sugar	16,522
R10D0	XaIRK	[D]Impaired fasting glycaemia	2,640
	44T2.	Fasting blood glucose (& level)	1,052

Table S39: HbA1c codes.

Read v2	CTV3	Description	Records
42W5.	XaPbt	Haemoglobin A1c level - International Fede...	378,381
42W4.	XaERp	HbA1c level (DCCT aligned)	174,955
44TB.	X772q	Haemoglobin A1c level	112,070
42W..	XE24t	Hb. A1C - diabetic control	28,342
42c..	XaCES	HbA1 - diabetic control	3,909
42W2.	42W2.	Hb. A1C 7-10% - borderline	2,147
42W1.	42W1.	Hb. A1C <7% - good control	1,722
66Ae0	XaWP9	HbA1c (haemoglobin A1c) target level - IFC...	1,636
42WZ.	42WZ.	Hb. A1C - diabetic control NOS	1,275
	X80U3	Glycosylated haemoglobin	852

Table S40: 2–hour oral glucose tolerance test codes.

Read v2	CTV3	Description	Records
44V..	XE25Z	Glucose tolerance test	21,381
7P172	XE25Z	Glucose tolerance test	16,651
C11y2	X40Jh	Impaired glucose tolerance	4,757
44g6.	XaEOV	120 minute plasma glucose level	3,530
R102.	R102.	[D]Glucose tolerance test abnormal	2,472
R10E.	XaInl	[D]Impaired glucose tolerance	2,174
44V1.	44V1.	Glucose tolerance test normal	654
44TH.	XaENB	120 minute blood glucose level	631
44f6.	XaEOZ	120 minute serum glucose level	503
44V2.	44V2.	Glucose tol. test impaired	425

7.8. Drug prescriptions

Table S41: Anti–diabetic drug codes.

Drug	BNF	Read v2
Insulin	`060101`	`f1, f2, fw`
Antidiabetic drugs[1]	`060102`	`f3, f4, f5, ft`
Metformin[2]	`0601022`<br>`06010202`[3]<br>`0601020200`[3]	`f4`

[1] Non–insulin drugs including metformin. Read v2 codes correspond to SULFONYLUREAS, BIGUANIDES, GUAR and OTHER DRUGS USED IN DIABETES respectively.
[2] Metformin drugs excluding combination therapies.
[3] Non–standard BNF codes used in TPP data.

Table S42: Steroid drug codes.

Drug	BNF	Read v2
Steroid tablets/depot injections	`060302`	`fe`

Table S43: Statin drug codes. TPP BNF codes do not include chemical substance however statins appear to be recorded under the non–standard code `021204`. These records were also included.

Drug	BNF	Read v2
Rosuvastatin Calcium	`0212000AA`	`bxk`
Simvastatin	`0212000Y0`	`bxd`
Simvastatin & Ezetimibe	`0212000AC`	
Fenofibrate/Simvastatin	`0212000AJ`	
Atorvastatin	`0212000B0`	`bxi`
Cerivastatin	`0212000C0`	`bxj`
Fluvastatin Sodium	`0212000M0`	`bxg`
Lovastatin[1]	`0212000R0`	
Pravastatin Sodium	`0212000X0`	`bxe`

[1] Not present in Read v2 coding.

Table S44: Anti–hypertensive drug codes.

Drug	BNF	Read v2
Thiazides and related diuretics	020201	b2
includes Hydrochlorothiazide		bkC, bkI
Potassium–sparing diuretics and aldosterone antagonists	020203	b4
Potassium sparing diuretics and compounds	020204	b5
Beta–adrenoceptor blocking drugs	0204	bd
Vasodilator antihypertensive drugs	020501	be
Bosentan		bkA
Sitaxentan		bkE
Ambrisentan		bkG
Macitentan		bkK
Centrally–acting antihypertensive drugs	020502	bf
includes Rauwolfia Alkaloids		bk2
Adrenergic neurone blocking drugs	020503	bg
Other adrenergic neurone blocking drugs	020508	
Alpha–adrenoceptor blocking drugs	020504	bh
Metirosine		bk1
Renin–angiotensin system drugs	020505	bi[1]
Losartan		bk3
includes Valsartan		bk4, bkD, bkL
Irbesartan		bk5
Trandolapril[2]		bk6
Candesartan		bk7
Telmisartan		bk8
Eprosartan		bk9
includes Olmesartan		bkB, bkC, bkH, bkI
Aliskiren[3]		bkF
Azilsartan		bkJ
Calcium–channel blockers	020602	
includes Verapamil		bb3, bk6
includes Amlodipine		blb, bkD, bkH, bkI
Diltiazem		bl5
Nicardipine		bl7
Nifedipine		bl8
Isradipine		bla
Felodipine		blc
Lacidipine		ble
Nisoldipine		blg
Lercanidipine		blh

[1] Primarily ACE–inhibitors.
[2] ACE–inhibitor not included under bi.
[3] Renin–inhibitor.

Table S45: Atypical anti–psychotic drug codes. IMPORTANT: these codes do not identify prescription records in TPP data (see table S46).

Drug	BNF	Read v2
Amisulpride	`0402010A0`	`d4t`
Aripiprazole	`0402010AD` `0402020AD`[1]	`d4v`
Clozapine	`0402010C0`	`d4l`
Lurasidone	`0402010AI`	`d4y`
Olanzapine	`040201060` `0402020AC`[1]	`d4r`
Paliperidone	`0402010AE` `0402020AB`[1]	`d4w`
Quetiapine	`0402010AB`	`d4s`
Risperidone	`040201030` `0402020AA`[1]	`d4p`
Sertindole	`040201050`	`d4q`
Zotepine	`0402010AC`	`d4u`

[1] Depot injections.

Table S46: Atypical anti–psychotic drug search terms. TPP BNF codes do not include chemical substance therefore drugs were identified by searching the drug name field in all TPP records with BNF code starting `0402` for these generic and brand terms. Fuzzy matching was used e.g. `"Abilify"` or `"abilify-10mg"` matched but `"Stabilify"` would not.

Drug	Search terms (case insensitive)
Amisulpride	`"Amisulpride", "Solian"`
Aripiprazole	`"Abilify", "Aripiprazole"`
Clozapine	`"Clozapine", "Clozaril", "Denzapine", "Zaponex"`
Lurasidone	`"Latuda", "Lurasidone"`
Olanzapine	`"Arkolamyl", "Olanzapine", "Zalasta", "Zypadhera", "Zyprexa"`
Paliperidone	`"Invega", "Paliperidone", "Trevicta", "Xeplion"`
Quetiapine	`"Alaquet", "Atrolak", "Biquelle", "Brancico", "Ebesque", "Mintreleq", "Psyquet", "Quetiapine", "Seotiapim", "Seroquel", "Sondate", "Tenprolide", "Zaluron"`
Risperidone	`"Risperdal", "Risperidone"`
Sertindole	`"Serdolect", "Sertindole"`
Zotepine	`"Zoleptil", "Zotepine"`

8. UK BIOBANK CODING

Demographic inputs were taken from UK Biobank visit data as these were generally unavailable in the linked EHR data. Relevant biomarkers measured by UK Biobank were used to augment those extracted from the EHR data. The fields used are summarised in table S47. Mapping for ethnicity and smoking are summarised in tables S48 to S50.

The codes used to identify self–reported conditions are in table S51. Self–reported medications were identified from field 20003 by searching for drug names (both generic and brand names). The search terms are available at https://github.com/philipdarke/ehr-codesets. Any self–reported medications matching a steroid term were excluded if they also included the terms "eye", "ear" or "cream" as the aim was to identify "regular steroid tablets" as under the QDiabetes–2018 model.

Table S47: UK Biobank fields used in the analysis.

	UK Biobank field
**Demographic**	
Age[1]	34, 52
Sex	31
Townsend deprivation	189
Ethnicity	21000
**Anthropometric**	
Height	50
Weight	21002
Body mass index[2]	–
Waist circumference	48
**Biomarkers**	
HbA1c	30750
**Medical history**	
Smoking status and history	20116, 1249, 3456, 2887
Family history[3]	20107, 20110, 20111
Non–cancer medical conditions	20002, 20008
Current medications	20003
**Other**	
Date of UK Biobank assessment visit	53
Date of death	40000

[1] Estimated from year and month of birth fields.
[2] Calculated from height and weight fields.
[3] History was based on parents and siblings.

Table S48: Ethnicity codes and mapping for field 21000. The most recent self–reported ethnicity was used where data from multiple visits were available.

UKBB code[1]	UKBB description[1]	QDiabetes mapping
1	White	White or not recorded
1001	British	White or not recorded
1002	Irish	White or not recorded
1003	Any other white background	White or not recorded
2	Mixed	Other
2001	White and Black Caribbean	Caribbean
2002	White and Black African	Black African
2003	White and Asian	Other Asian
2004	Any other mixed background	Other
3	Asian or Asian British	Other Asian
3001	Indian	Indian
3002	Pakistani	Pakistani
3003	Bangladeshi	Bangladeshi
3004	Any other Asian background	Other Asian
4	Black or Black British	Other
4001	Caribbean	Caribbean
4002	African	Black African
4003	Any other Black background	Other
5	Chinese	Chinese
6	Other ethnic group	Other
-1	Do not know	White or not recorded
-3	Prefer not to answer	White or not recorded

[1] See https://biobank.ndph.ox.ac.uk/showcase/coding.cgi?id=1001

Table S49: Smoking status mapping.

Smoking status[1]	Past tobacco smoking[2]	Mapping
-3, 0	3, 4	Never smoked
-3, 1	1	Former smoker (level inferred from field 2887)
-3, 1	2	Former smoker (trivial)
2	-	Current smoker (level inferred from field 2456)

[1] Field 20116. See https://biobank.ndph.ox.ac.uk/showcase/coding.cgi?id=90.
[2] Field 1249. See https://biobank.ndph.ox.ac.uk/showcase/coding.cgi?id=100348.

Table S50: Smoking level mapping.

Number of daily cigarettes[1]	Mapping
<1	Trivial smoker
1-9	Light smoker
10-19	Moderate smoker
20+	Heavy smoker
Not provided	Assumed moderate smoker

[1] Field 2887 (current) or 3456 (former). See https://biobank.ndph.ox.ac.uk/showcase/coding.cgi?id=100353 and https://biobank.ndph.ox.ac.uk/showcase/coding.cgi?id=100355.

Table S51: Non–cancer condition mapping (field 20002).

Code[1]	Condition
1220, 1221, 1222, 1223	Diabetes (any type)
1065, 1072	Hypertension
1075	Myocardial infarction/heart attack
1074	Angina
1081, 1583	Stroke
1291	Bipolar affective disorder
1289	Schizophrenia
1350	Polycystic ovarian syndrome

[1] See https://biobank.ndph.ox.ac.uk/showcase/coding.cgi?id=6.

REFERENCES

[1] Helen Booth, D. Dedman, and Achim Wolf, "CPRD Aurum: frequently asked questions," Apr. 2019. [Online]. Available: https://cprd.com/sites/default/files/CPRD%20Aurum%20FAQs%20v2.0_2.pdf

[2] UK Biobank, "Resource 591: Primary care data," Sep. 2020. [Online]. Available: http://biobank.ndph.ox.ac.uk/showcase/refer.cgi?id=591

[3] S. Denaxas, A. D. Shah, B. A. Mateen, V. Kuan, J. K. Quint, N. Fitzpatrick, A. Torralbo, G. Fatemifar, and H. Hemingway, "A semi-supervised approach for rapidly creating clinical biomarker phenotypes in the UK Biobank using different primary care EHR and clinical terminology systems," *JAMIA Open*, vol. 3, no. 4, pp. 545–556, Dec. 2020. [Online]. Available: https://doi.org/10.1093/jamiaopen/ooaa047

[4] UK Biobank, "Resource 951: TPP COVID numeric reference codes," Mar. 2021. [Online]. Available: http://biobank.ndph.ox.ac.uk/showcase/refer.cgi?id=951

[5] J. Hippisley-Cox and C. Coupland, "Development and validation of QDiabetes-2018 risk prediction algorithm to estimate future risk of type 2 diabetes: cohort study," *BMJ*, vol. 359, Nov. 2017. [Online]. Available: https://doi.org/10.1136/bmj.j5019

[6] NHS Digital, "Technology Reference data Update Distribution." [Online]. Available: https://isd.digital.nhs.uk/trud3/user/guest/group/0/home

[7] D. Nagi, C. Hambling, and R. Taylor, "Remission of type 2 diabetes: a position statement from the Association of British Clinical Diabetologists (ABCD) and the Primary Care Diabetes Society (PCDS)," *British Journal of Diabetes*, vol. 19, no. 1, pp. 73–76, Jun. 2019, number: 1. [Online]. Available: https://doi.org/10.15277/bjd.2019.221

[8] National Institute for Health and Clinical Excellence, "Type 2 diabetes: prevention in people at high risk (PH38)," Sep. 2017. [Online]. Available: https://www.nice.org.uk/guidance/ph38

[9] World Health Organisation, "Definition and diagnosis of diabetes mellitus and intermediate hyperglycaemia," 2006. [Online]. Available: https://www.who.int/diabetes/publications/diagnosis_diabetes2006/en/

[10] L. J. Gray, N. A. Taub, K. Khunti, E. Gardiner, S. Hiles, D. R. Webb, B. T. Srinivasan, and M. J. Davies, "The Leicester Risk Assessment score for detecting undiagnosed Type 2 diabetes and impaired glucose regulation for use in a multiethnic UK setting," *Diabetic Medicine*, vol. 27, no. 8, pp. 887–895, 2010. [Online]. Available: https://doi.org/10.1111/j.1464-5491.2010.03037.x

[11] S. R. Barber, N. N. Dhalwani, M. J. Davies, K. Khunti, and L. J. Gray, "External national validation of the Leicester Self-Assessment score for Type 2 diabetes using data from the English Longitudinal Study of Ageing," *Diabetic Medicine*, vol. 34, no. 11, pp. 1575–1583, 2017, _eprint: https://onlinelibrary.wiley.com/doi/pdf/10.1111/dme.13433. [Online]. Available: https://doi.org/10.1111/dme.13433
